# Supplementary figures and images for: TranSynergy: Mechanism-driven interpretable deep neural network for the synergistic prediction and pathway deconvolution of drug combinations
Source: PLoS Comput Biol. 2021 Feb 12;17(2):e1008653. doi: 10.1371/journal.pcbi.1008653 (PMC7906476; doi:10.1371/journal.pcbi.1008653)

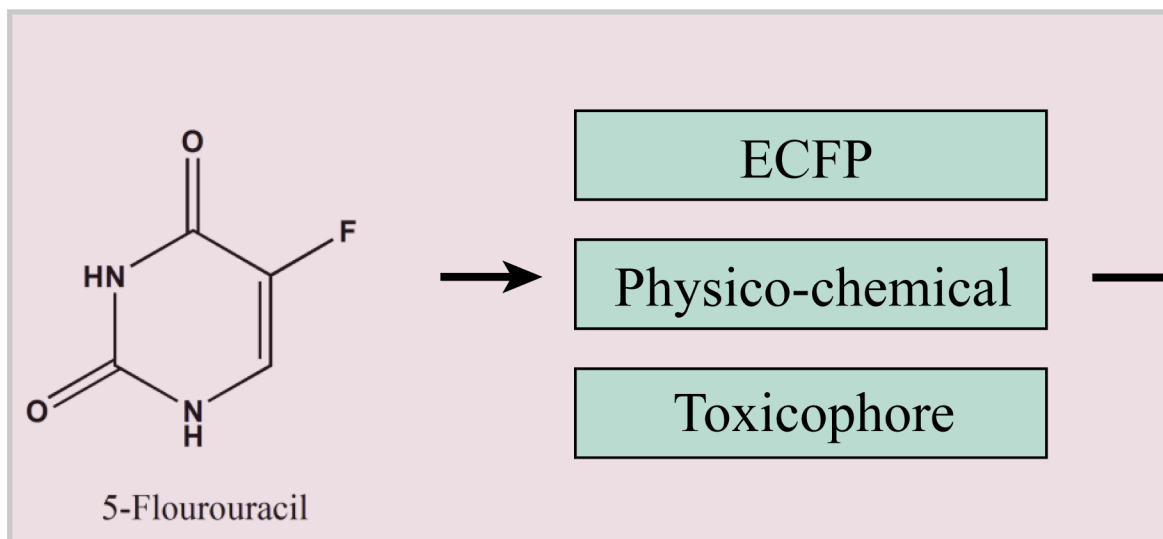

Dimension reduction

Transformer

Fully Connected

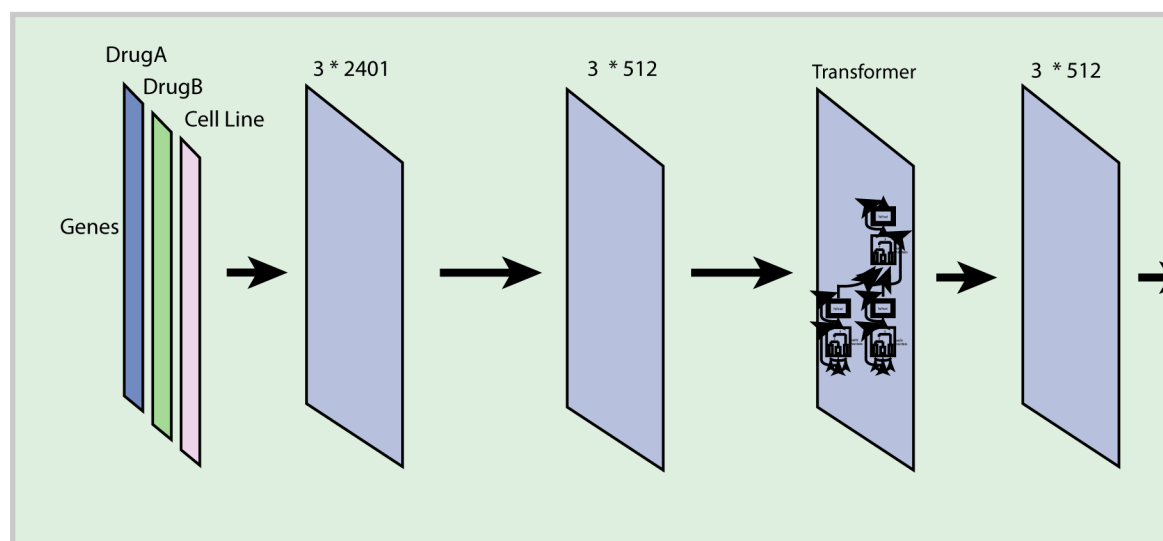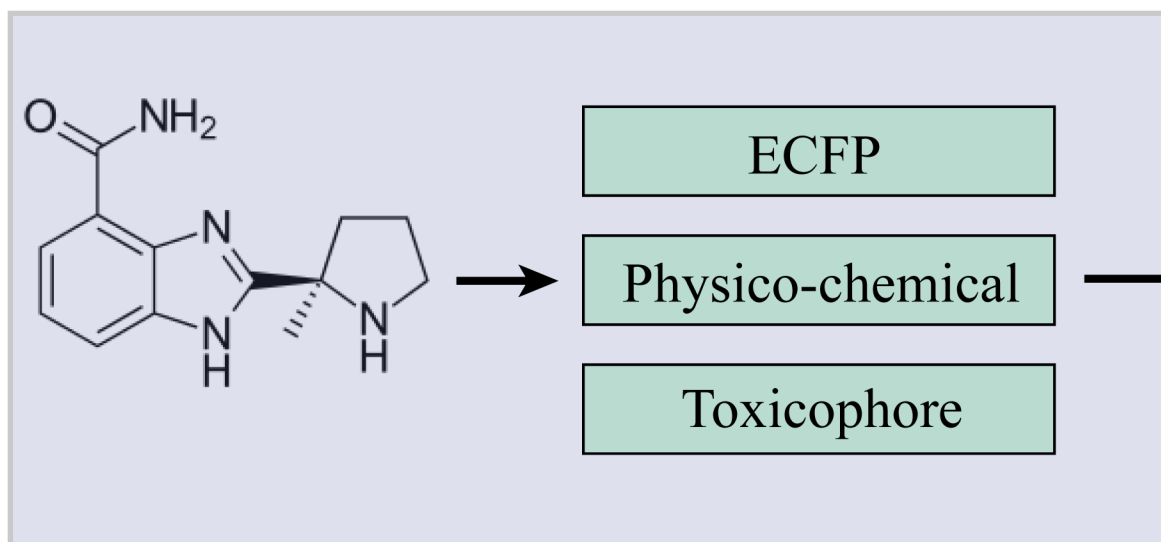

Supplement: S1 Fig — Extra drug features, including ECFP, physico-chemical and toxicophore features were concatenated with the output of the Transformer and input into the last fully connected component. These chemical features are the same as those used in deepSynergy. (PDF) [file pcbi.1008653.s007.pdf]

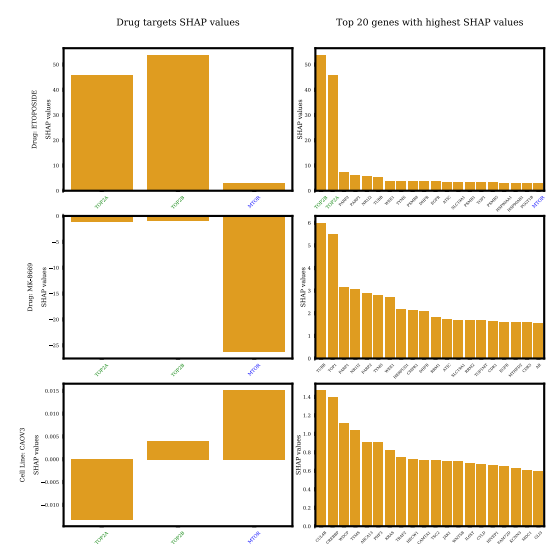

Supplement: S3 Fig — The left panel shows the SHAP values of drug targets, while the right panel shows the SHAP values of 20 genes with the most significant impact. (PDF) [file pcbi.1008653.s009.pdf]

Drug targets SHAP values

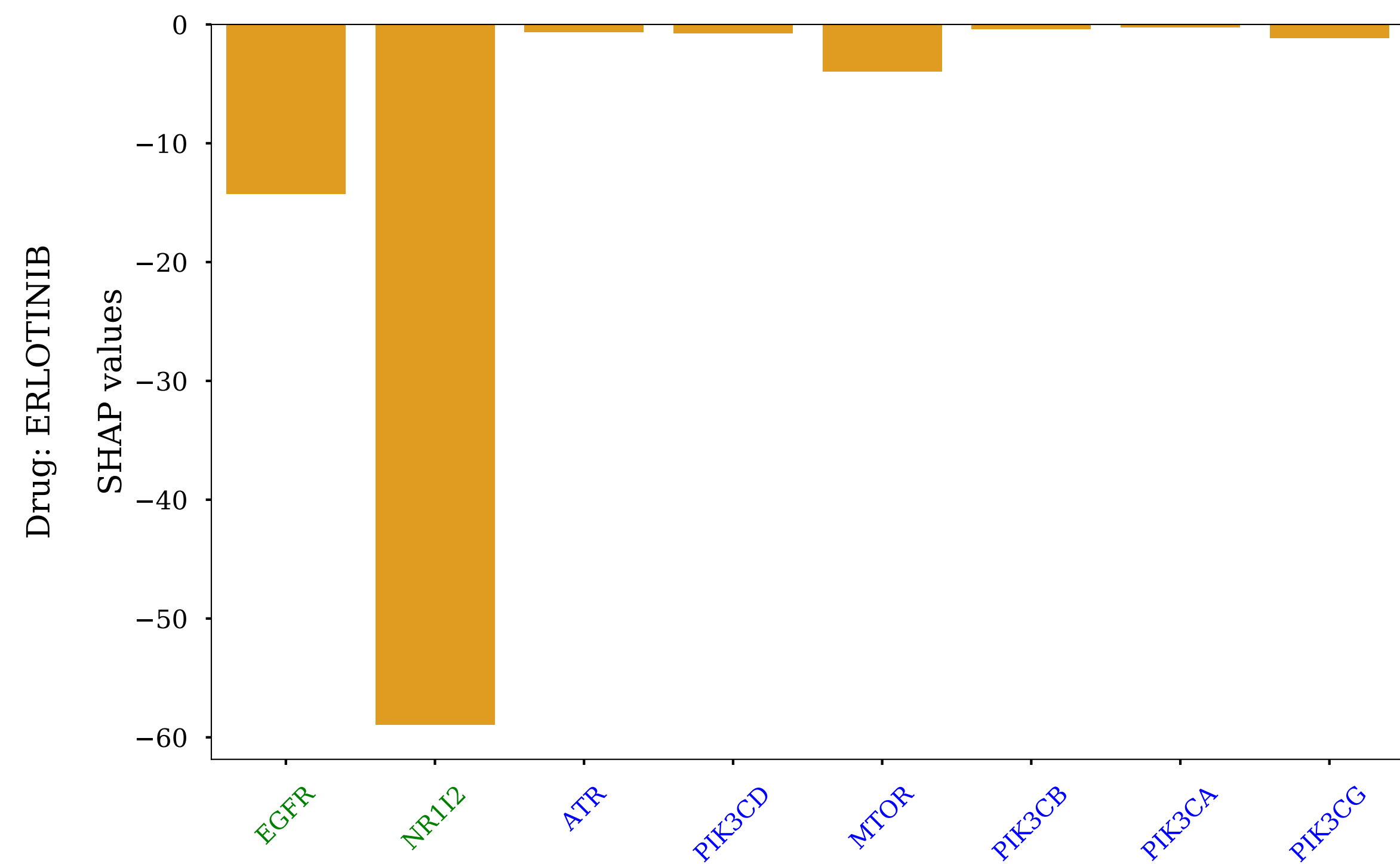

Top 20 genes with highest SHAP values

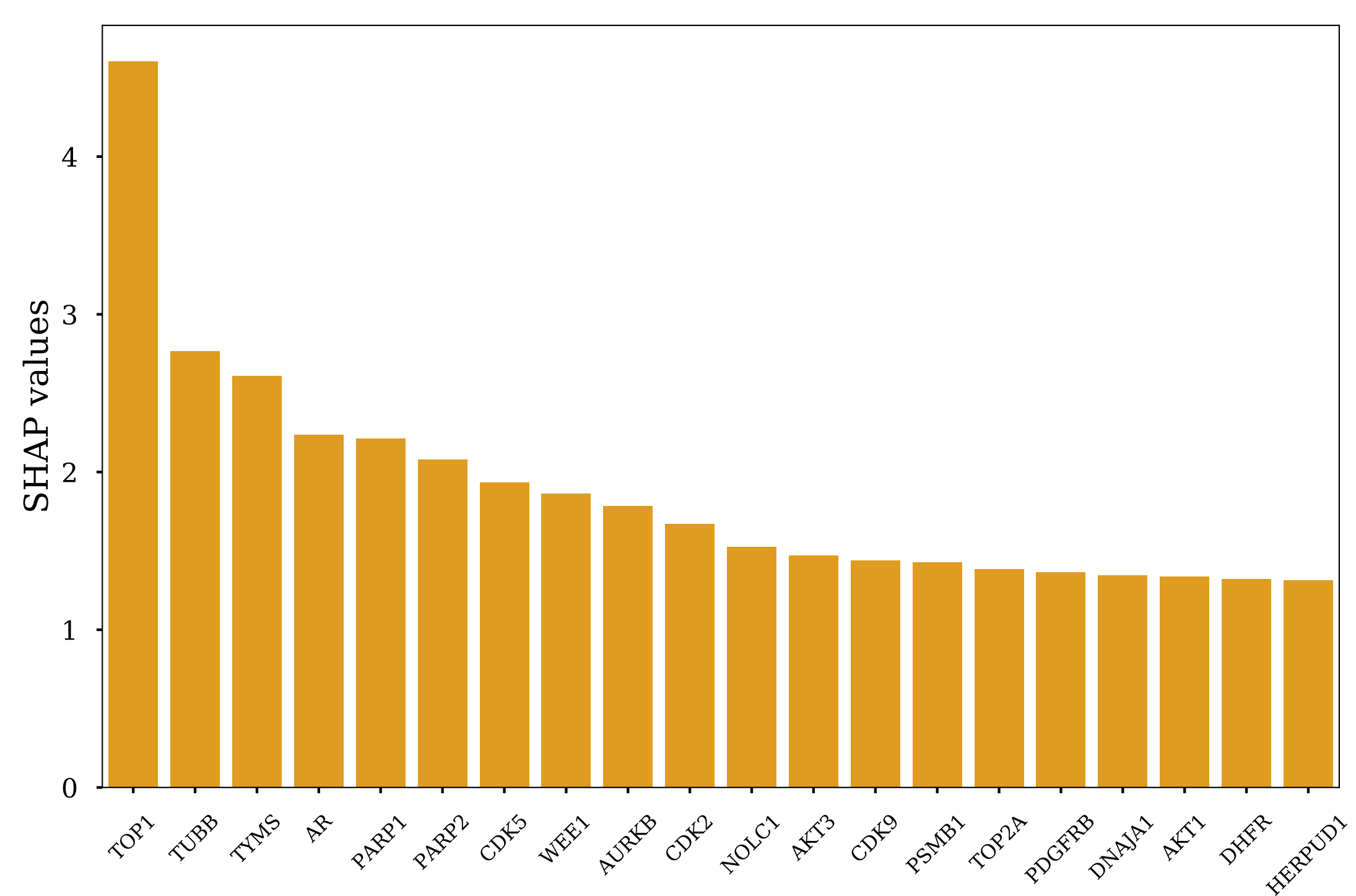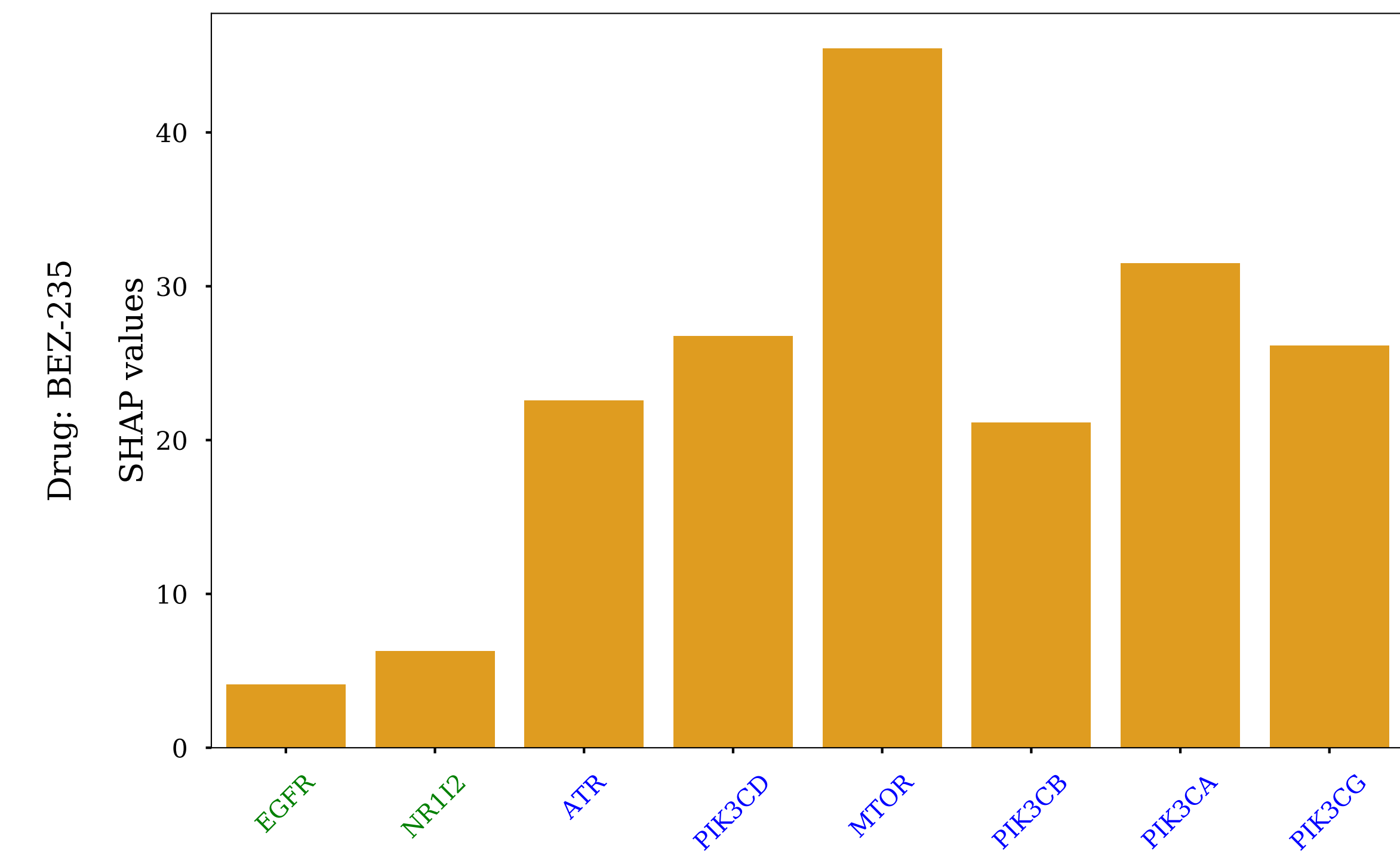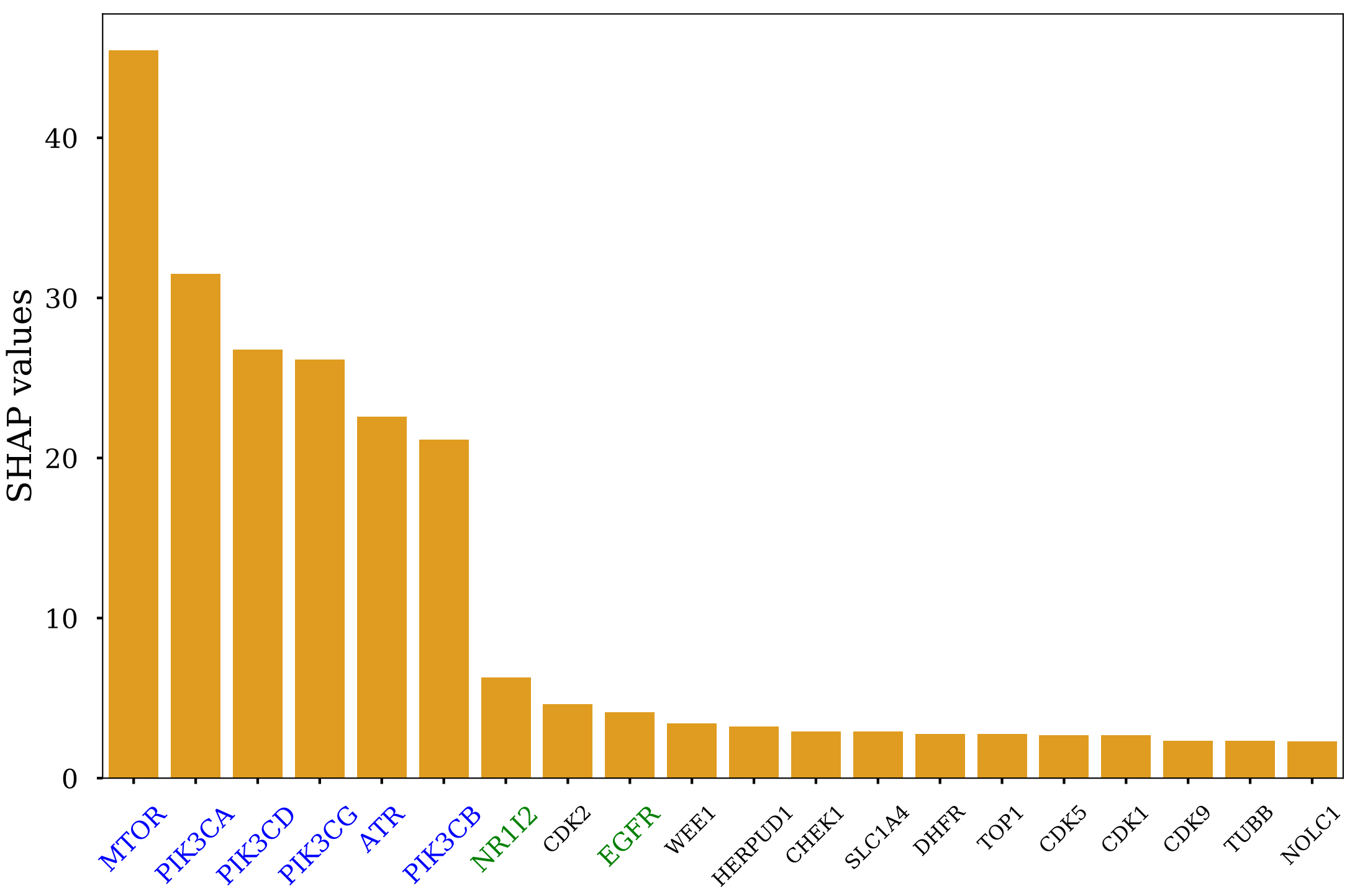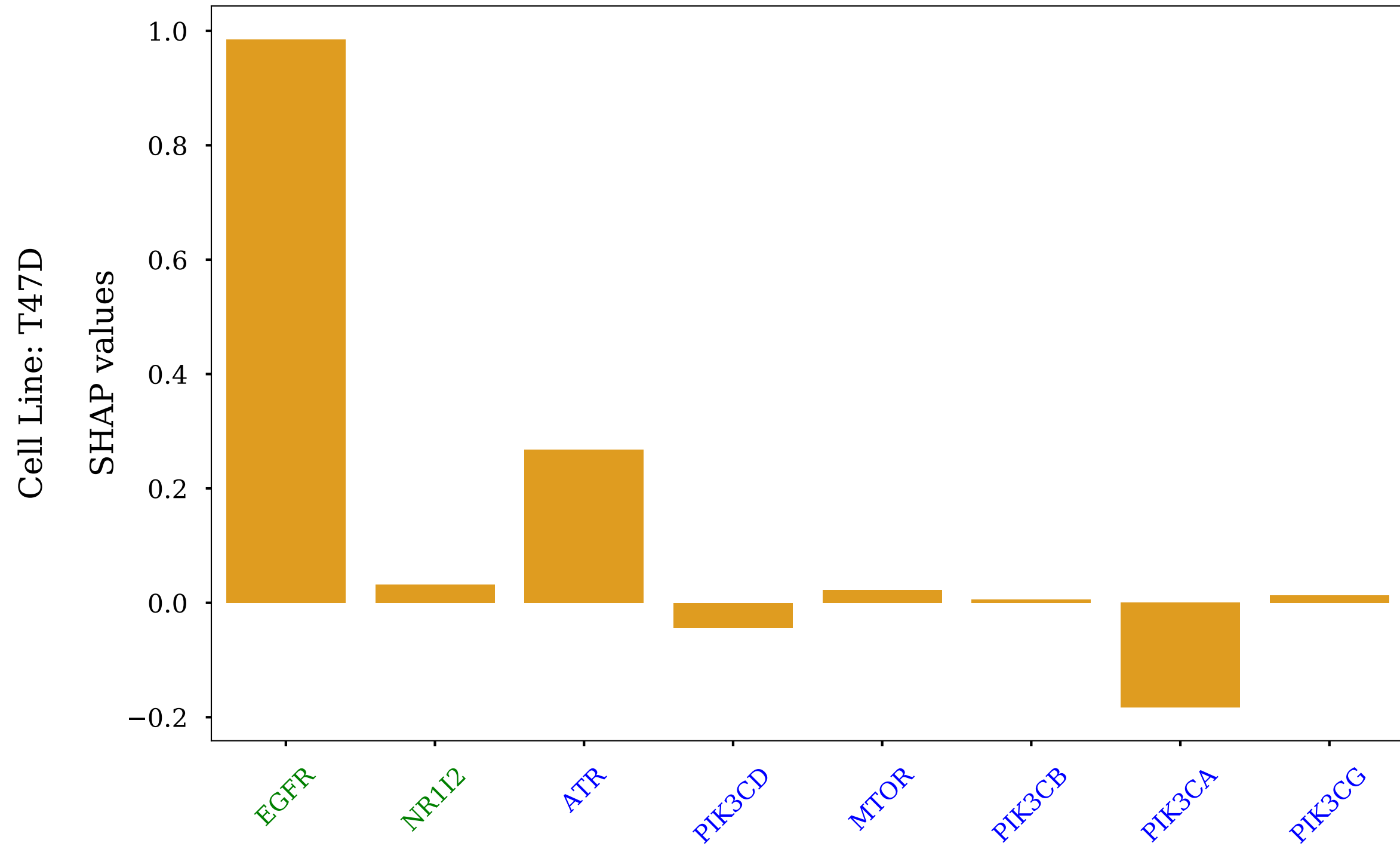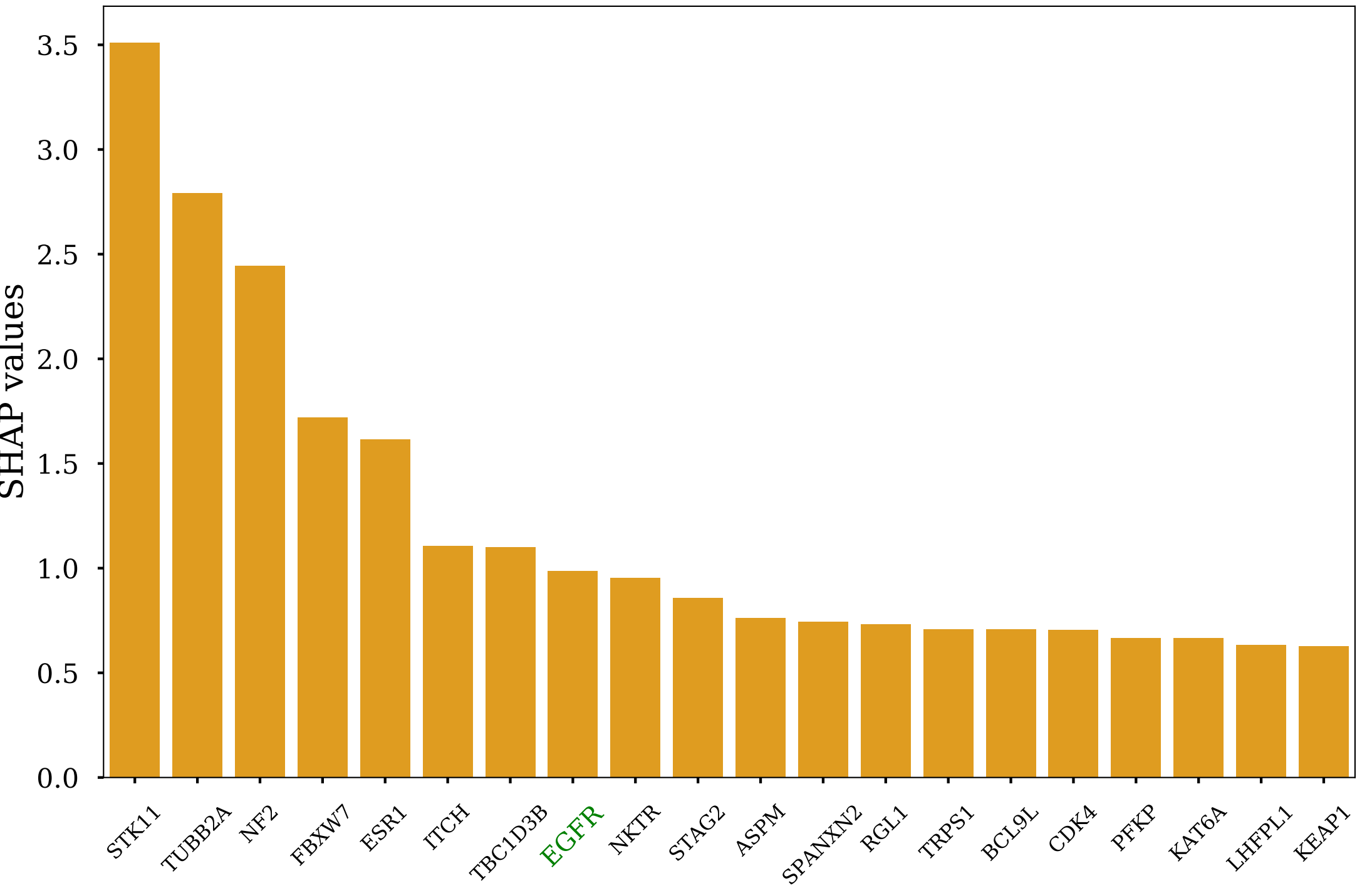

Supplement: S4 Fig — The left panel shows the SHAP values of drug targets, while the right panel shows the SHAP values of 20 genes with the most significant impact. (PDF) [file pcbi.1008653.s010.pdf]

Drug targets SHAP values

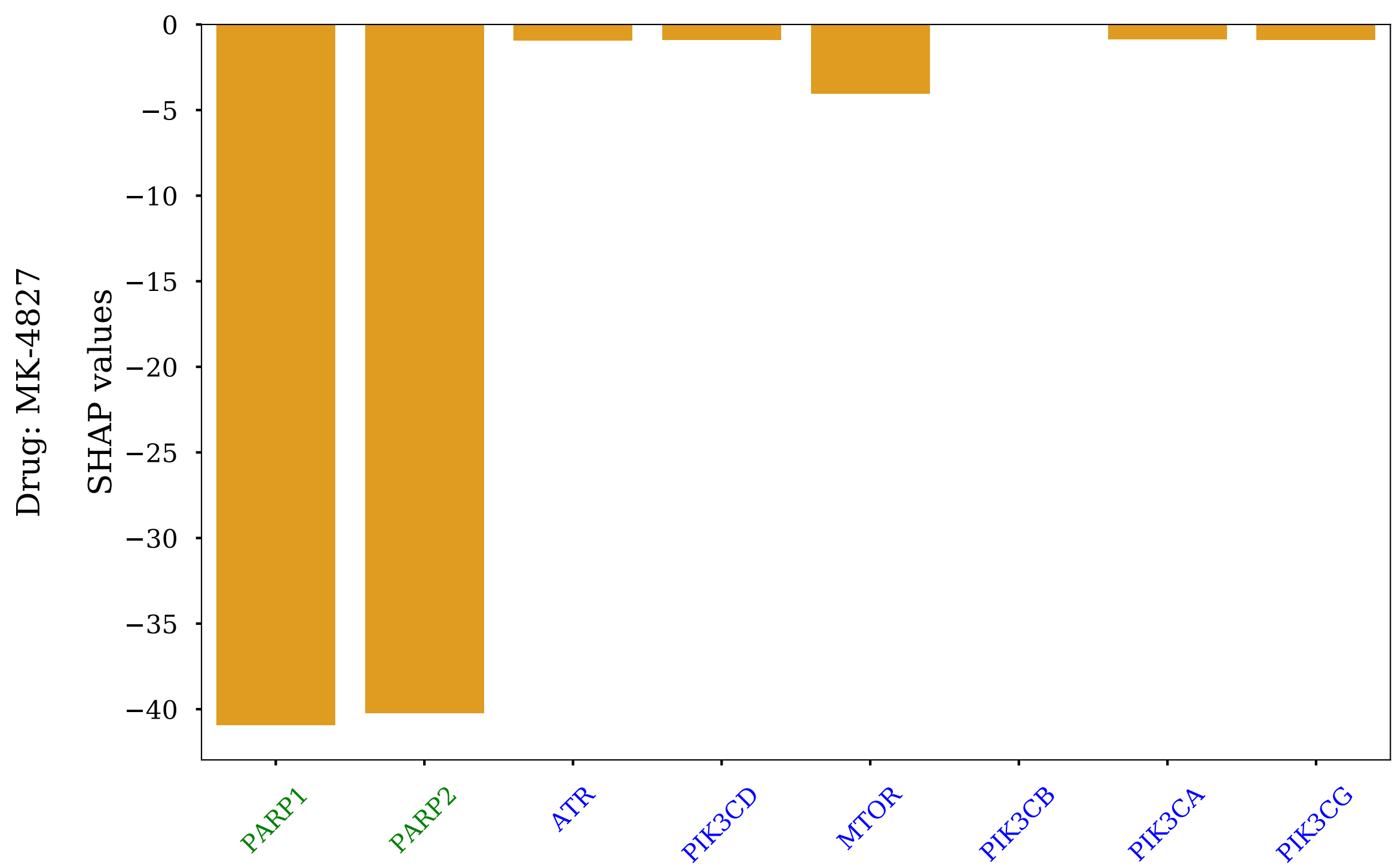

Top 20 genes with highest SHAP values

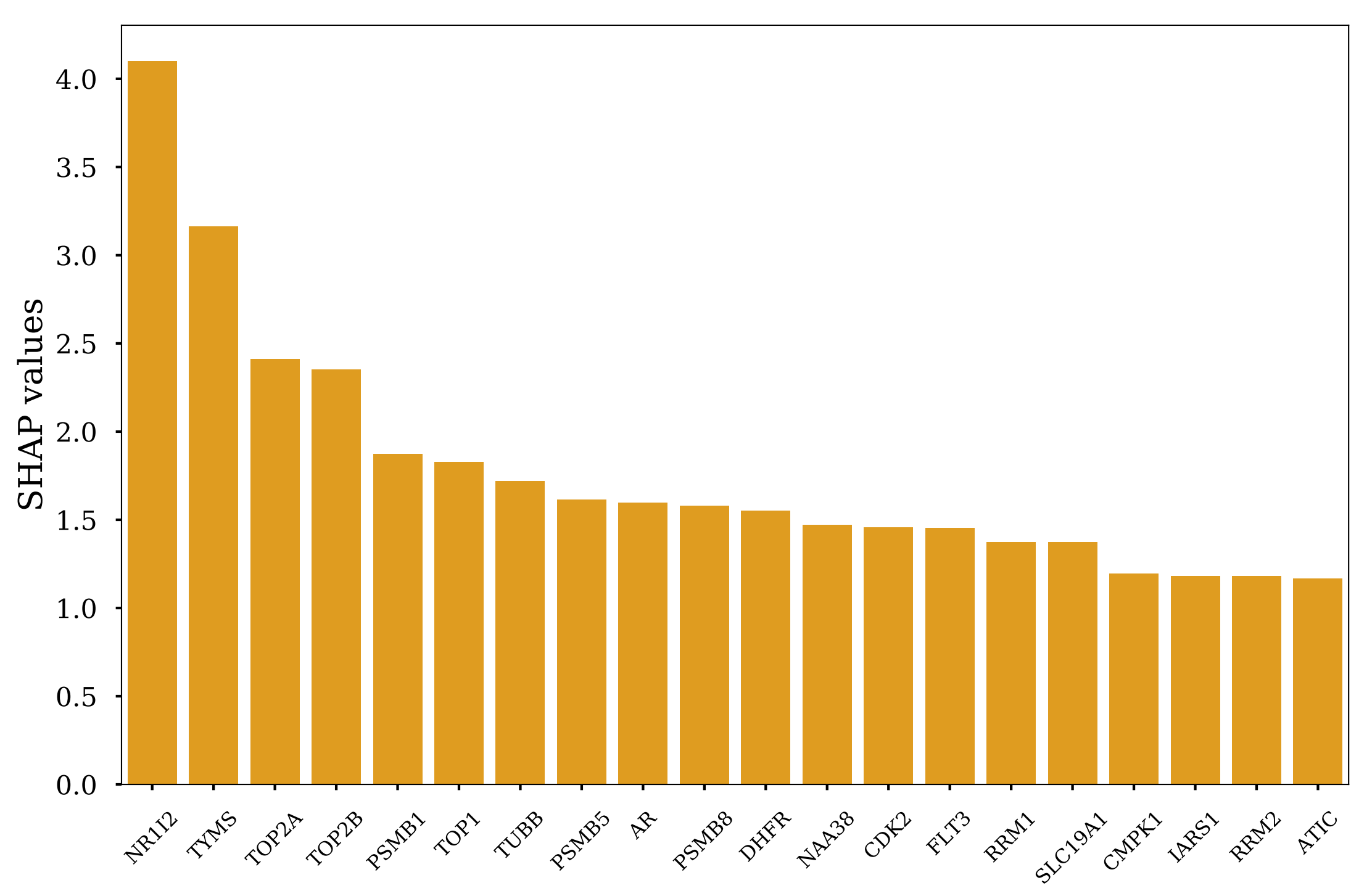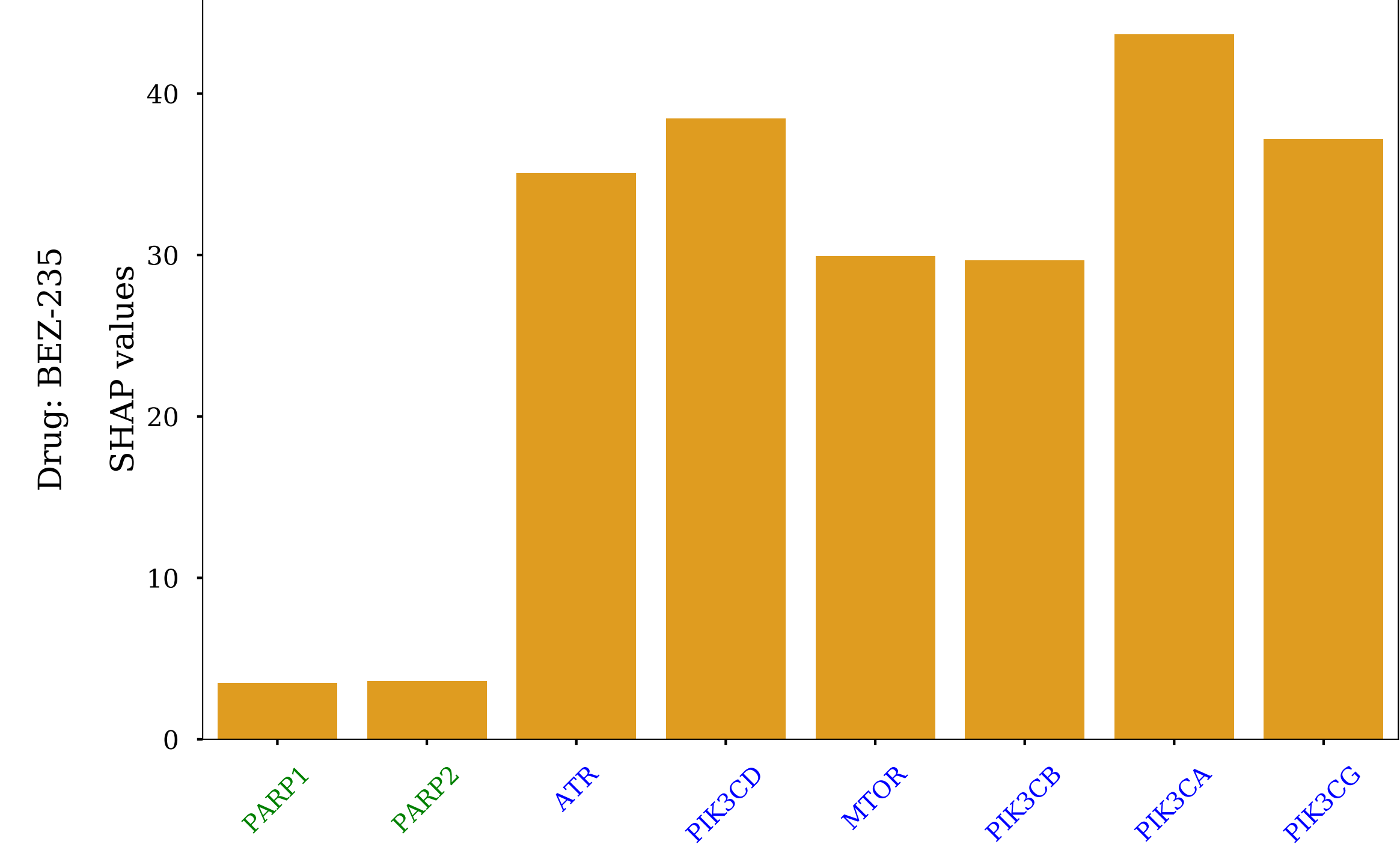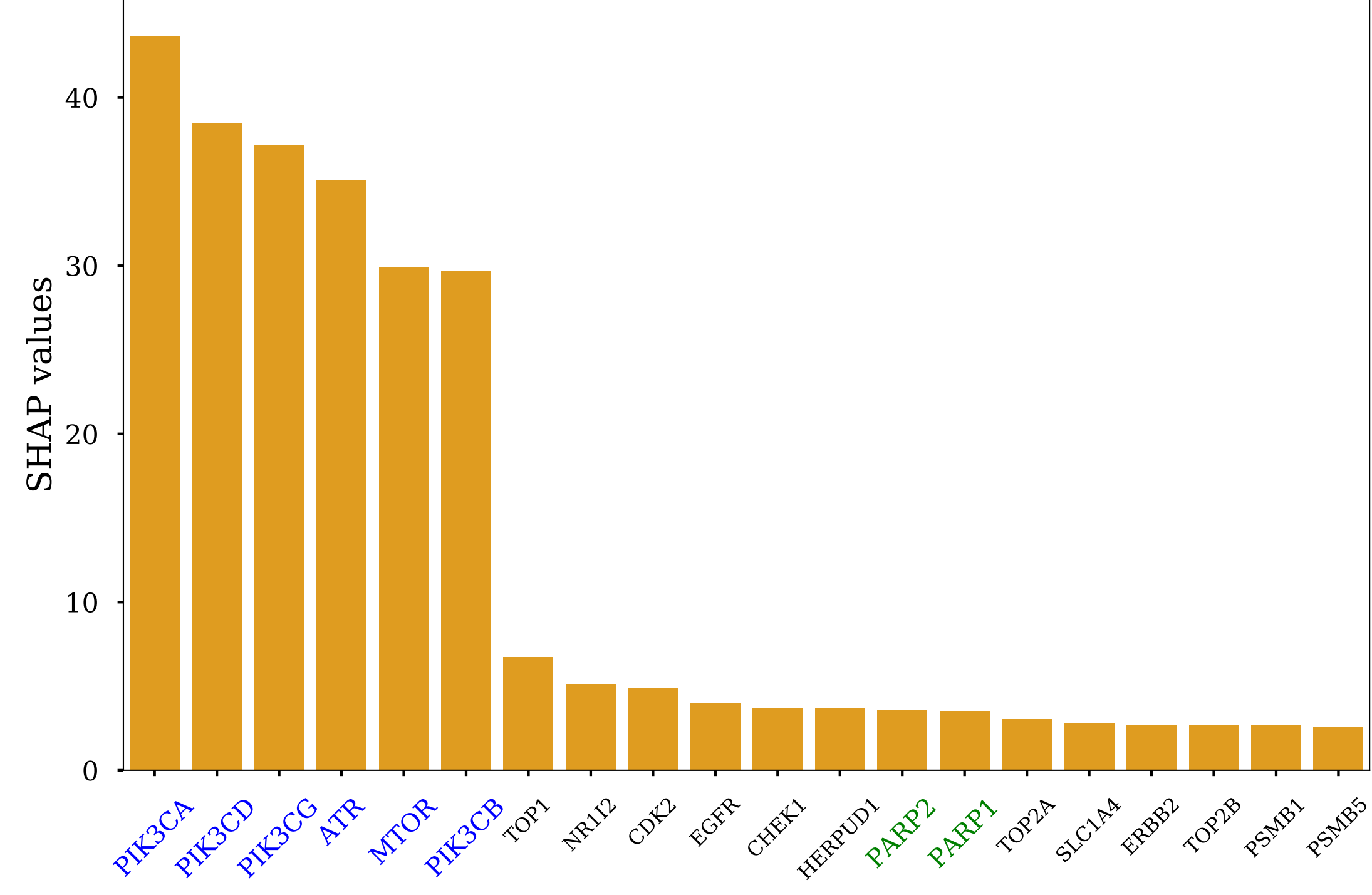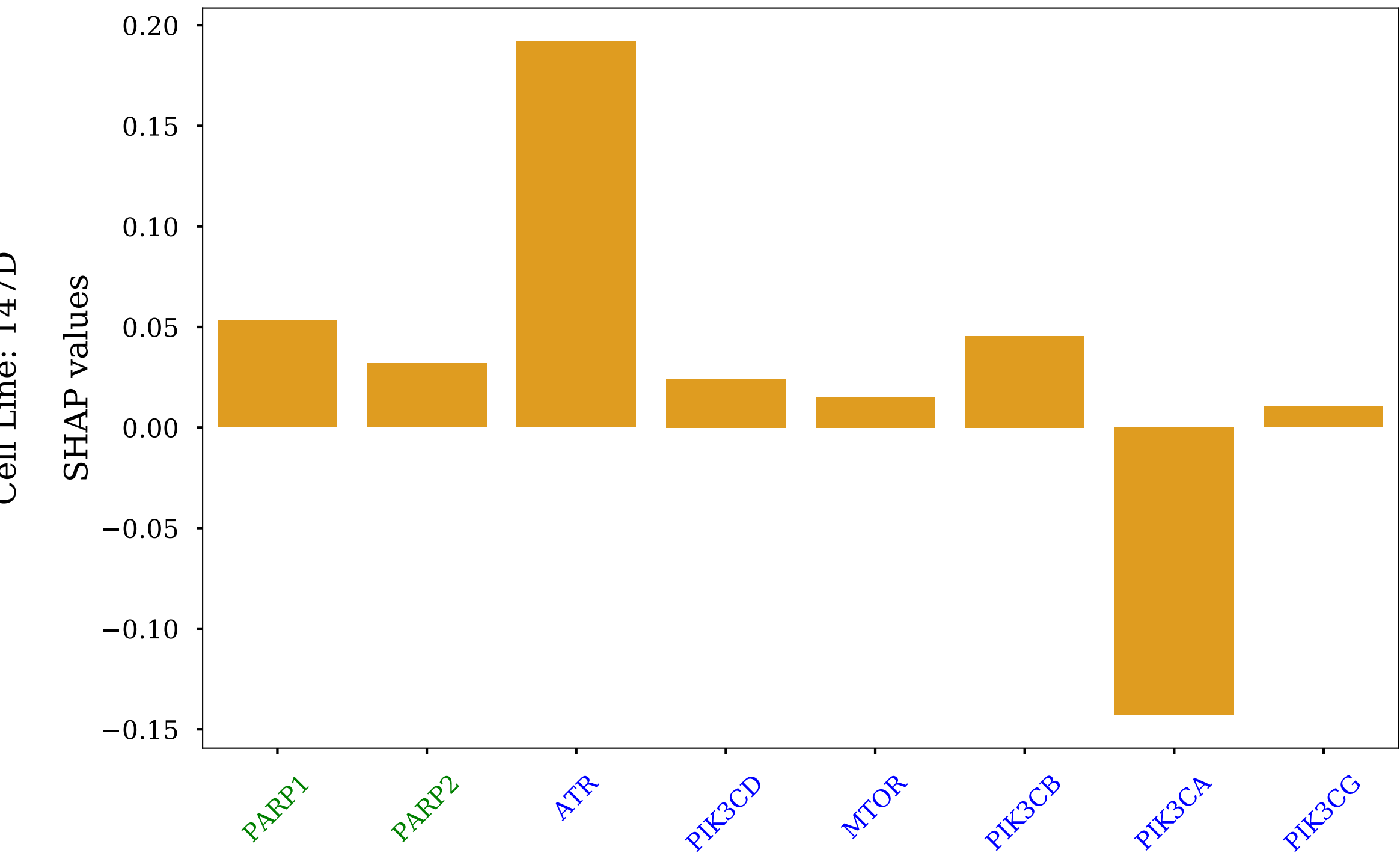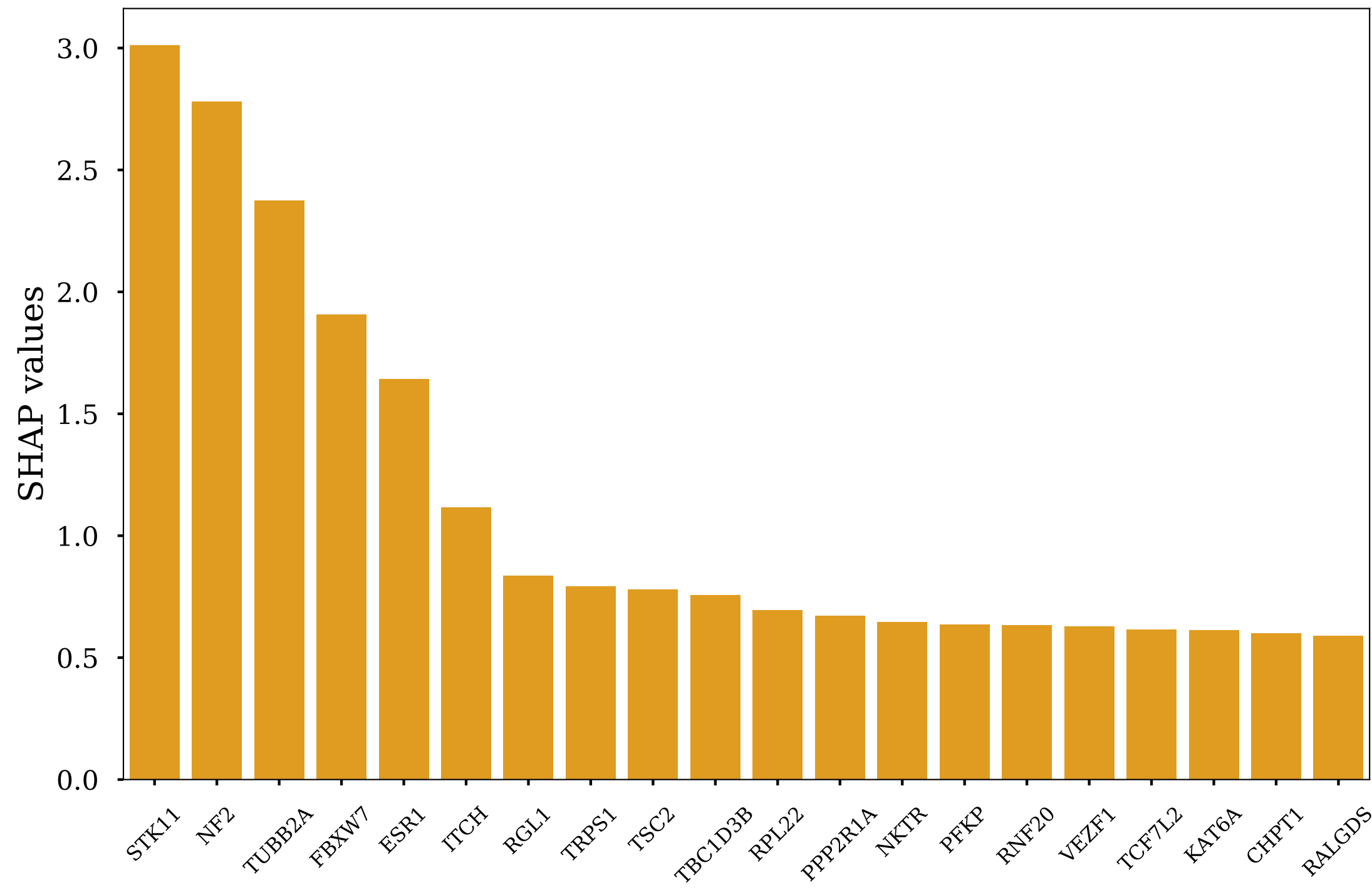

Supplement: S5 Fig — The left panel shows the SHAP values of drug targets, while the right panel shows the SHAP values of 20 genes with the most significant impact. (PDF) [file pcbi.1008653.s011.pdf]

Drug targets SHAP values

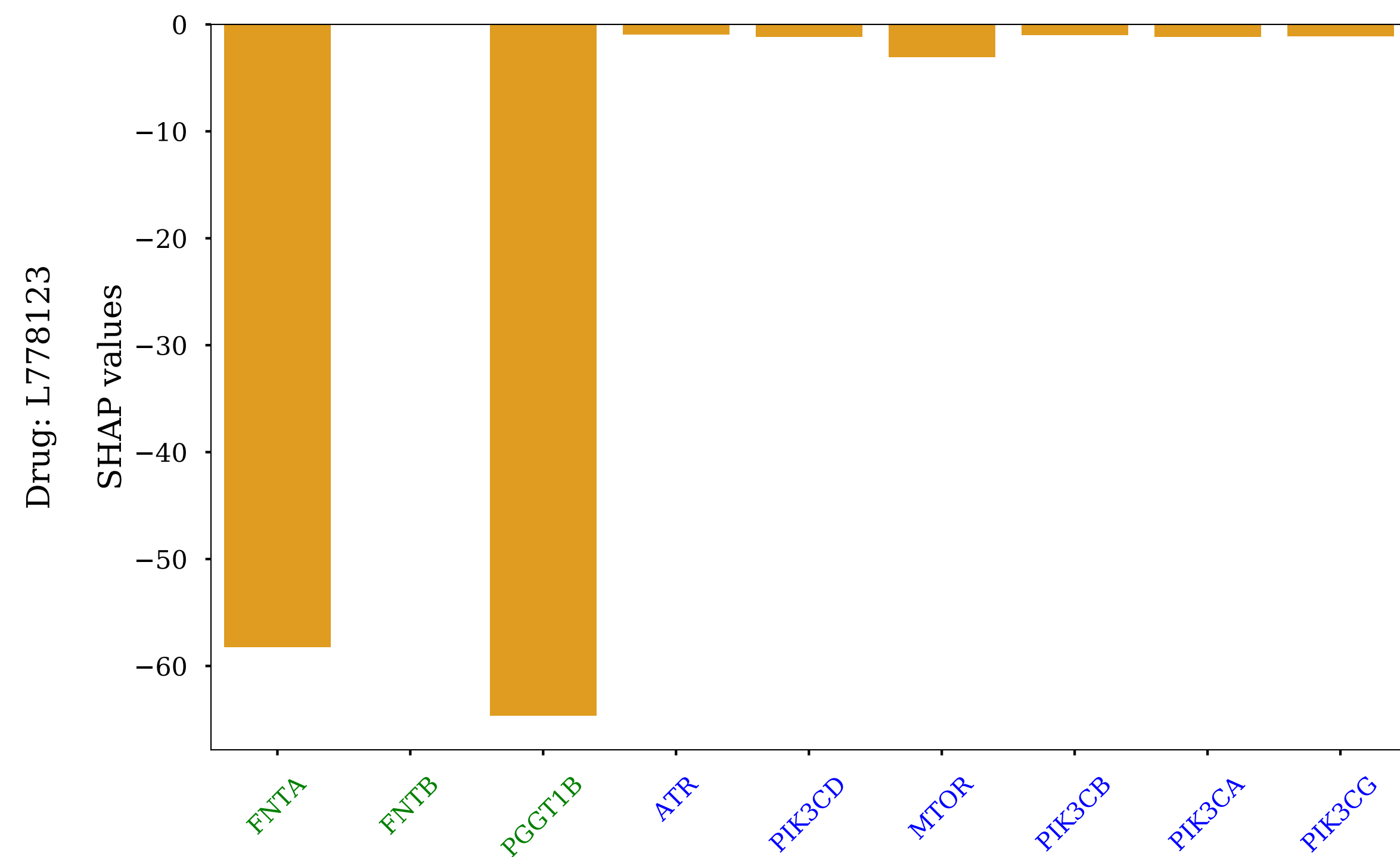

Top 20 genes with highest SHAP values

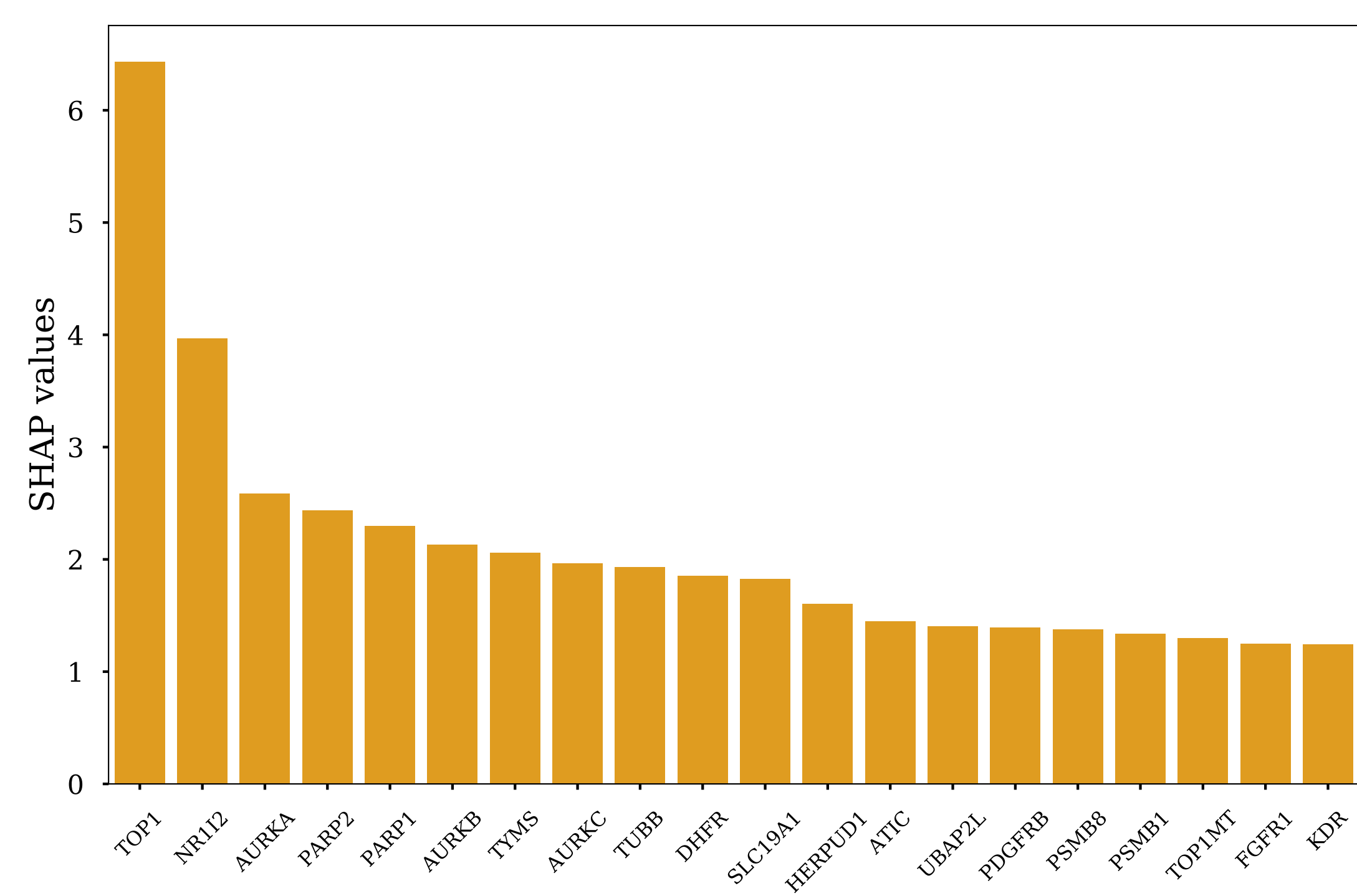

Drug: BEZ-235

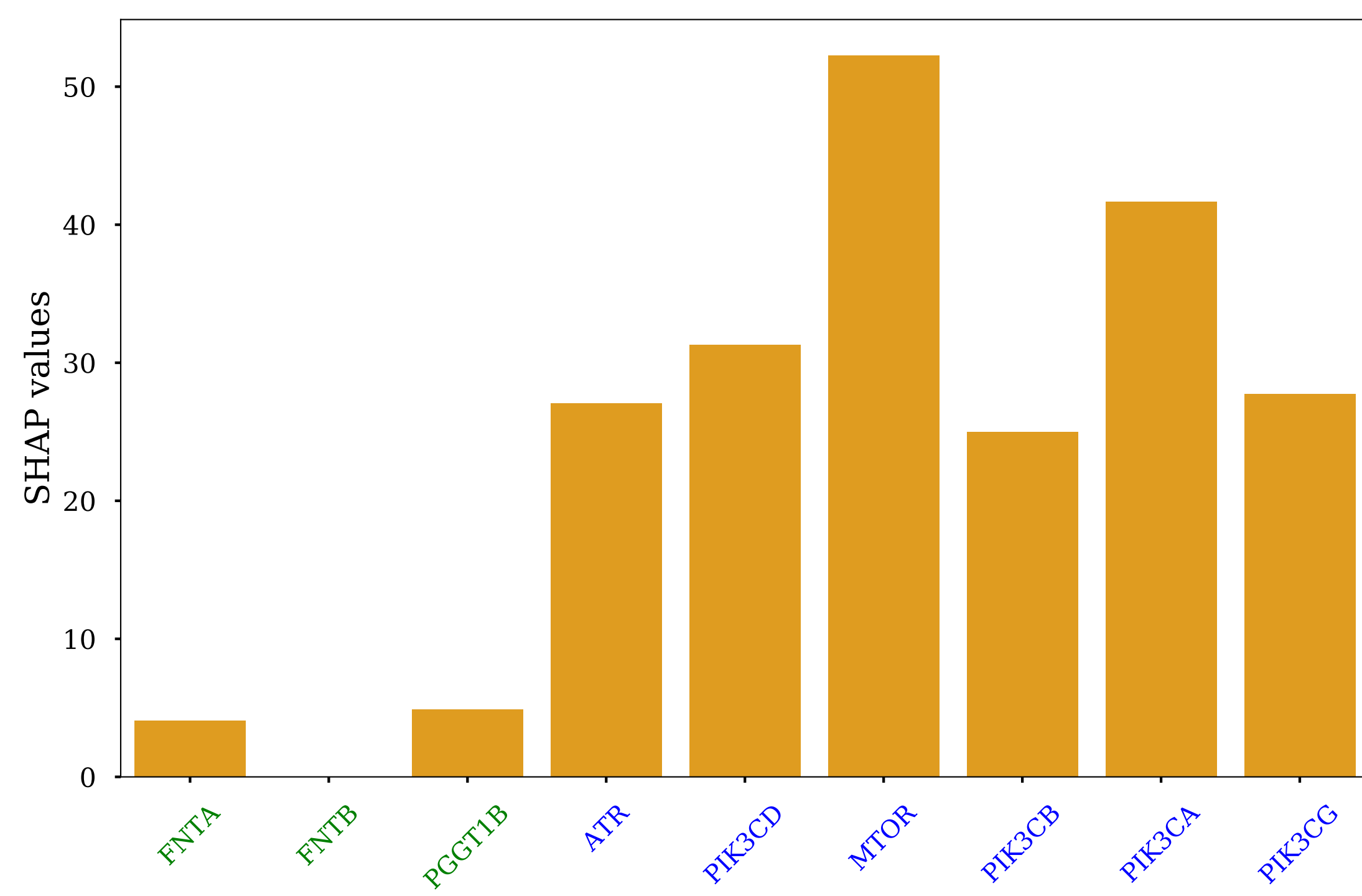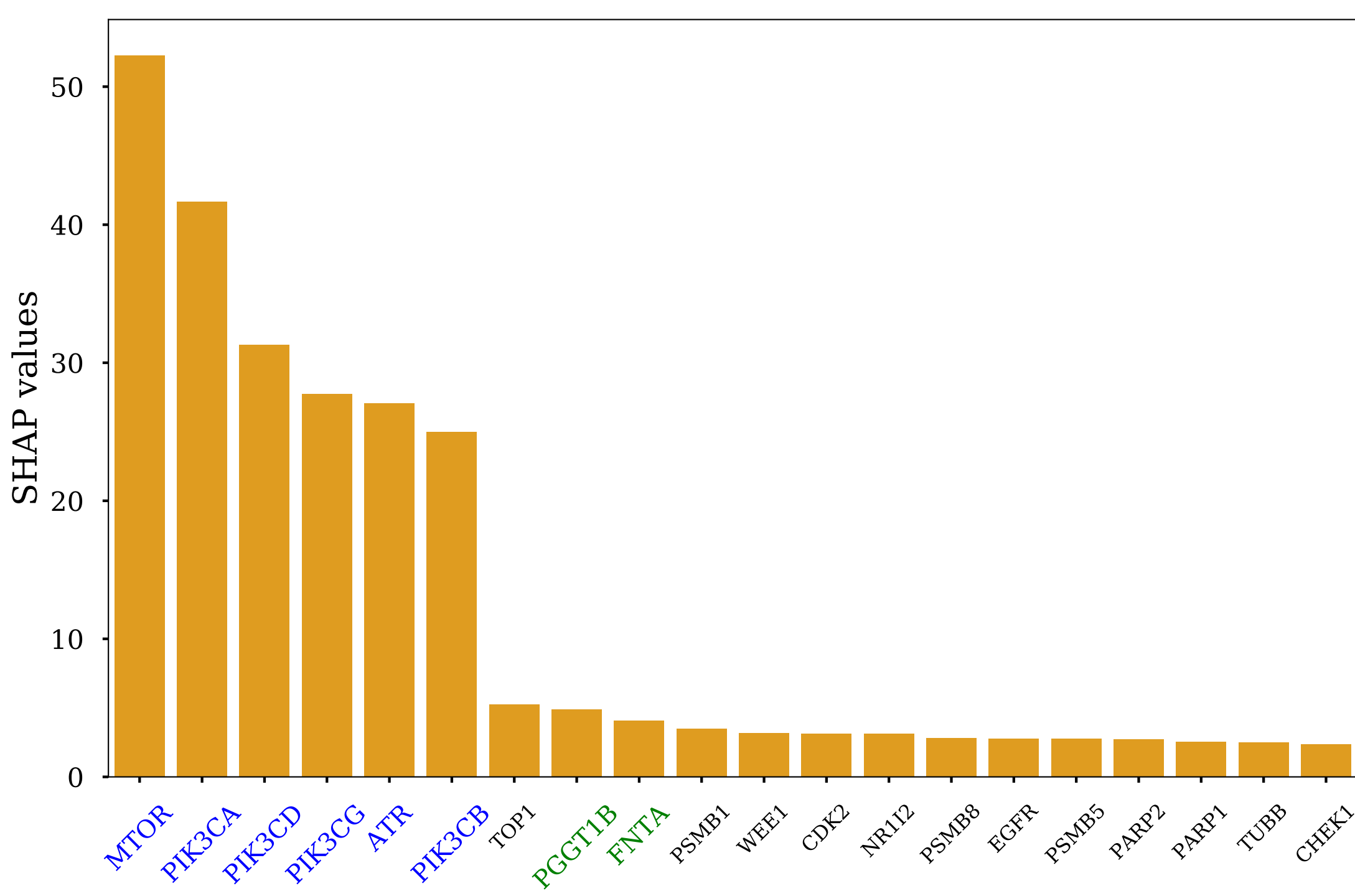

Cell Line: T47D

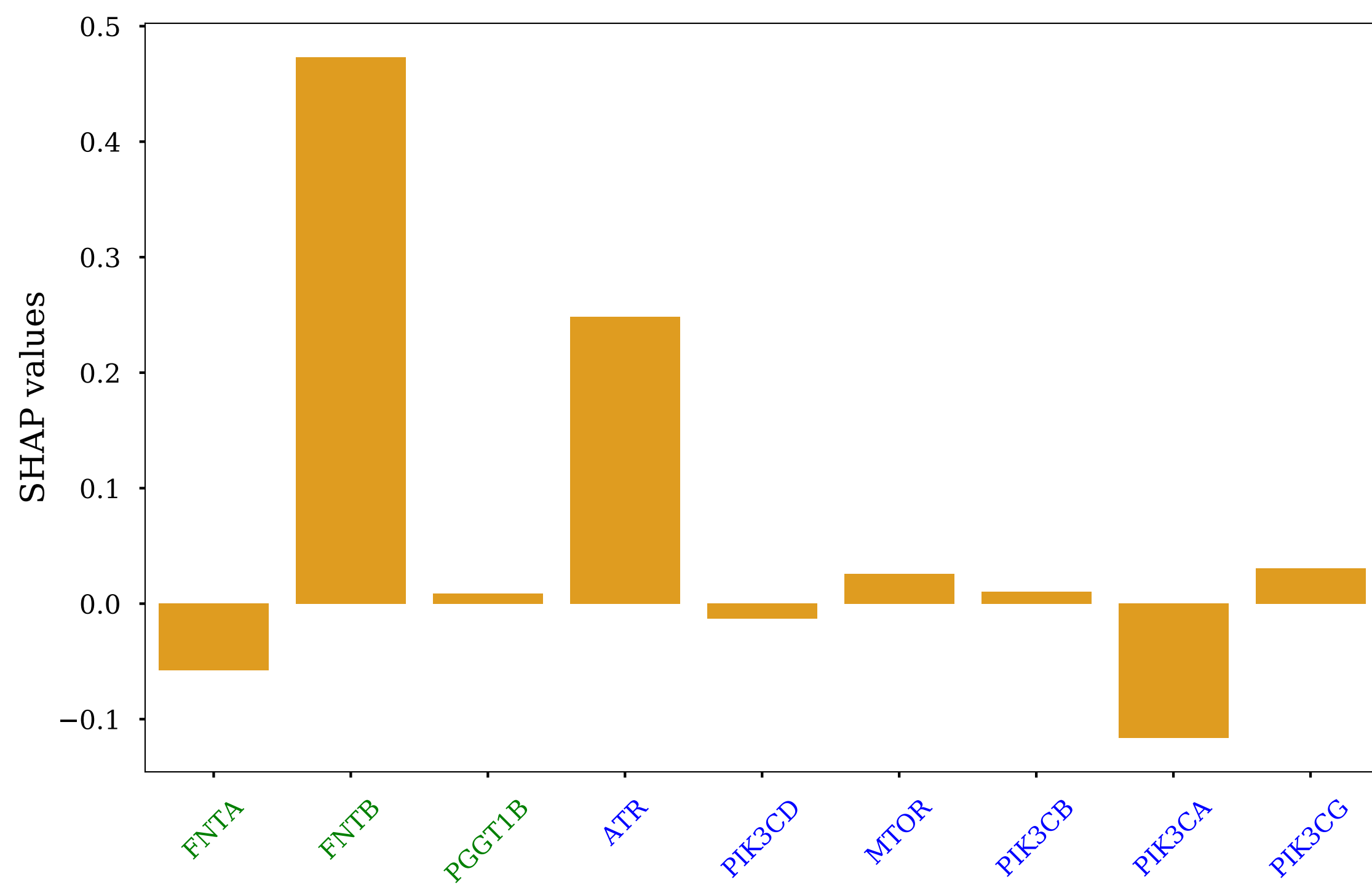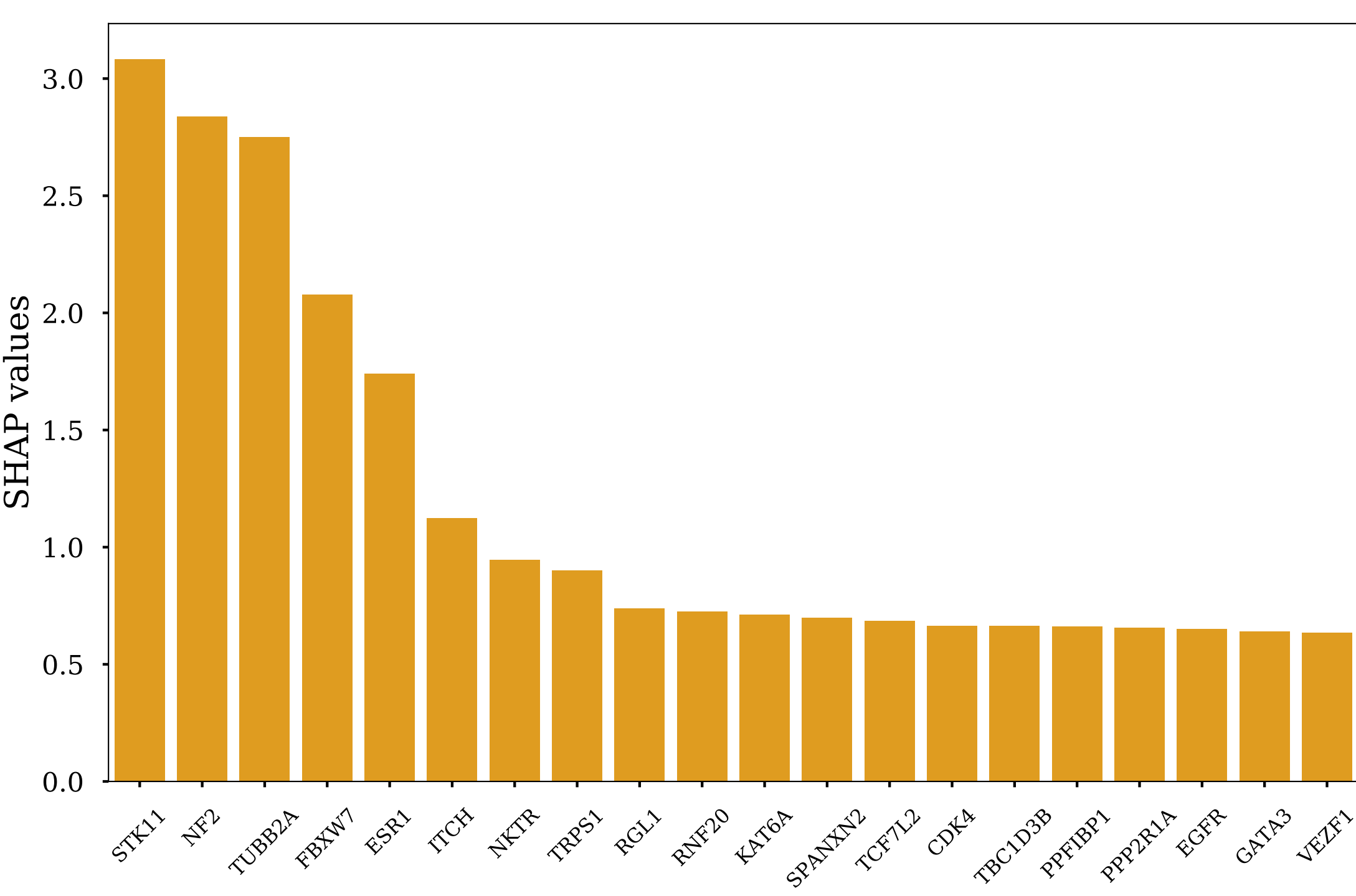

Supplement: S6 Fig — The left panel shows the SHAP values of drug targets, while the right panel shows the SHAP values of 20 genes with the most significant impact. (PDF) [file pcbi.1008653.s012.pdf]

### Drug targets SHAP values

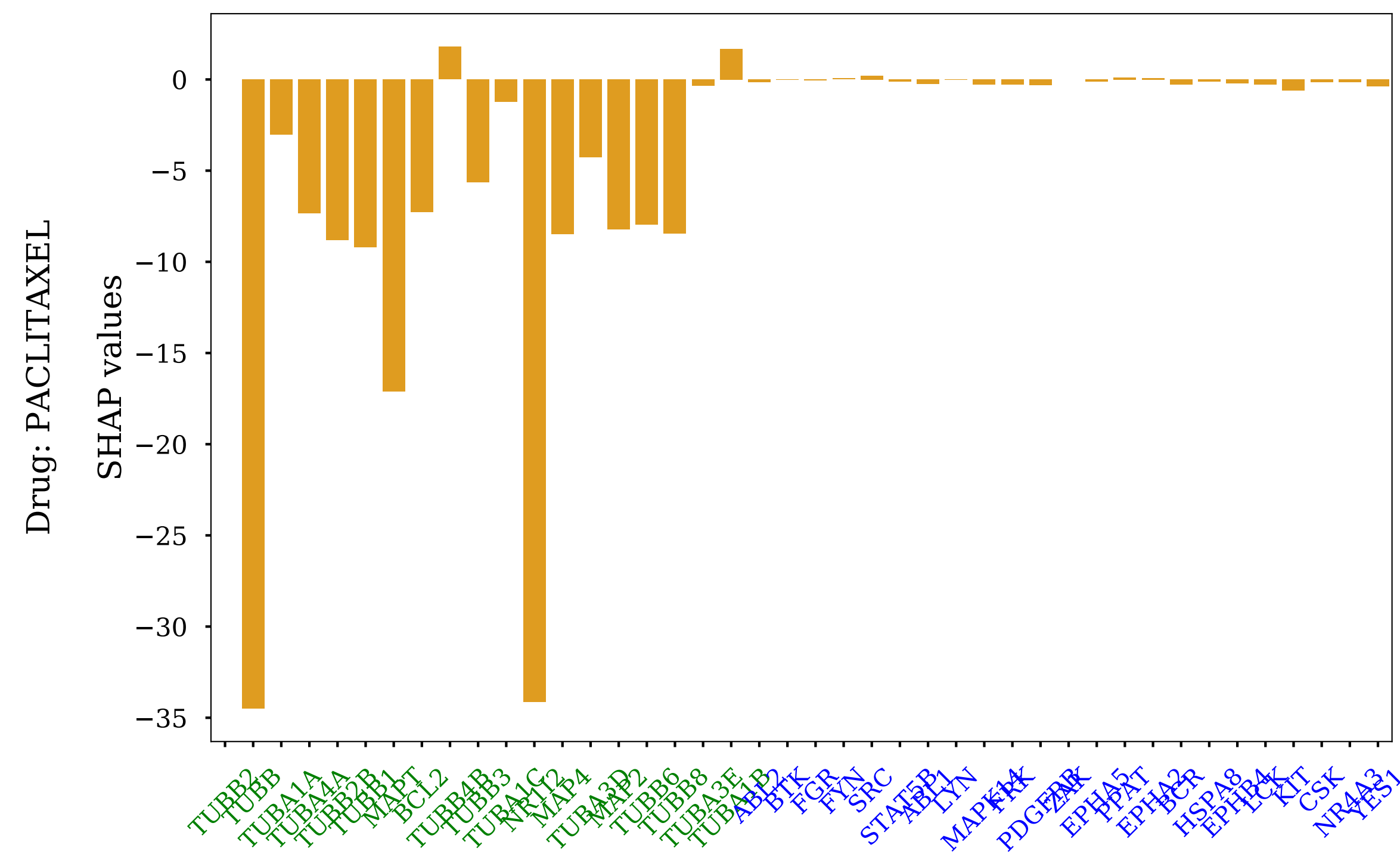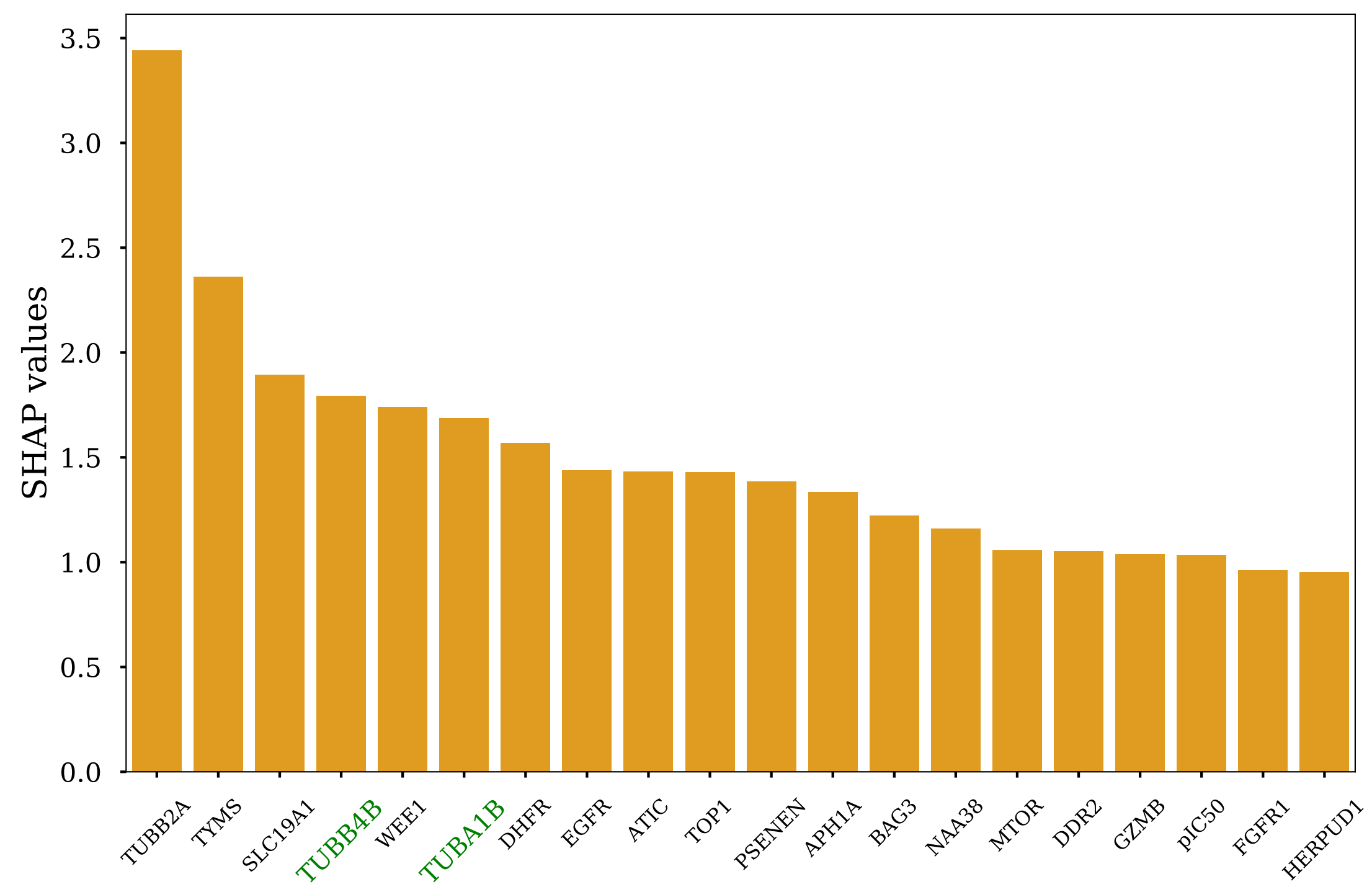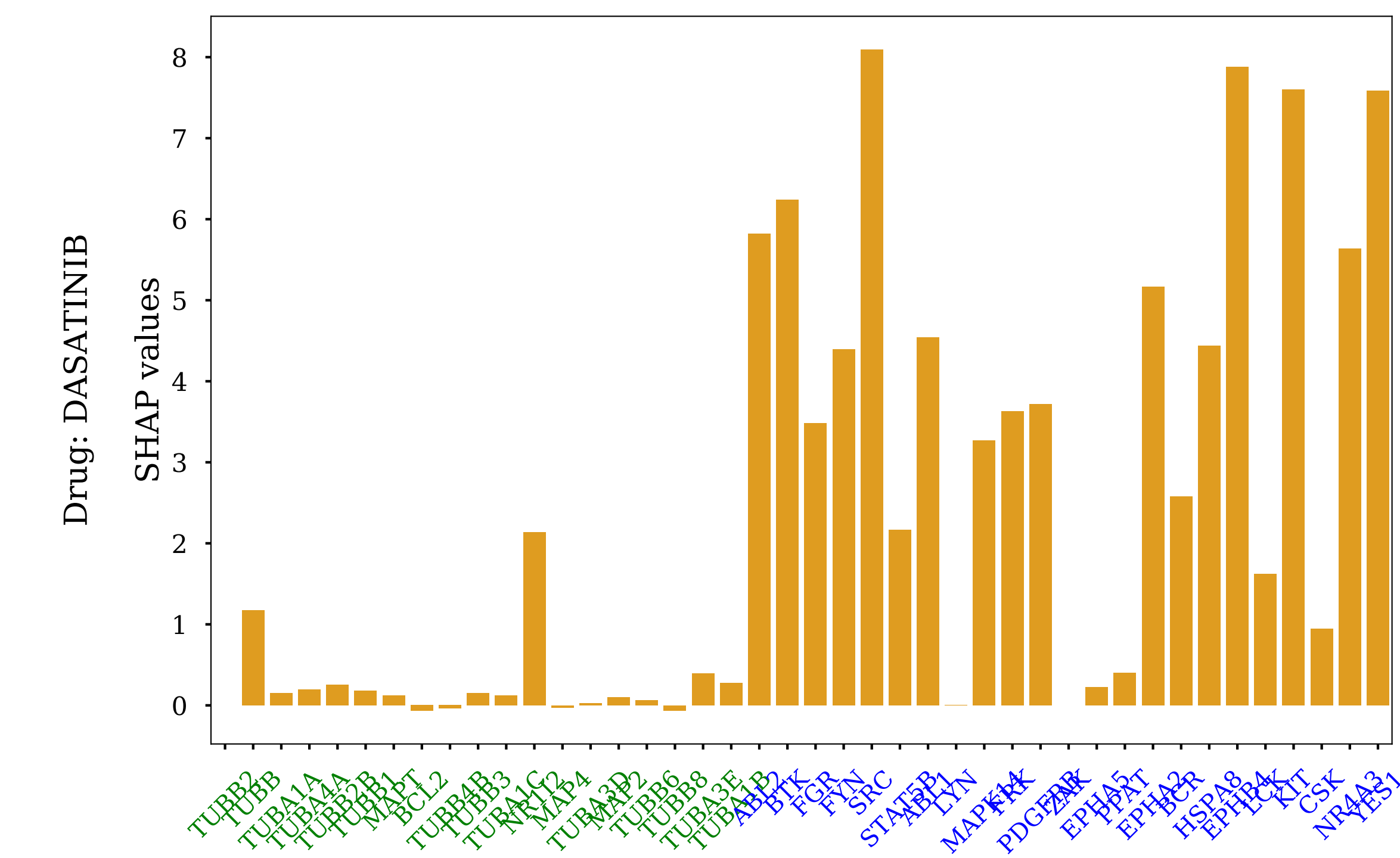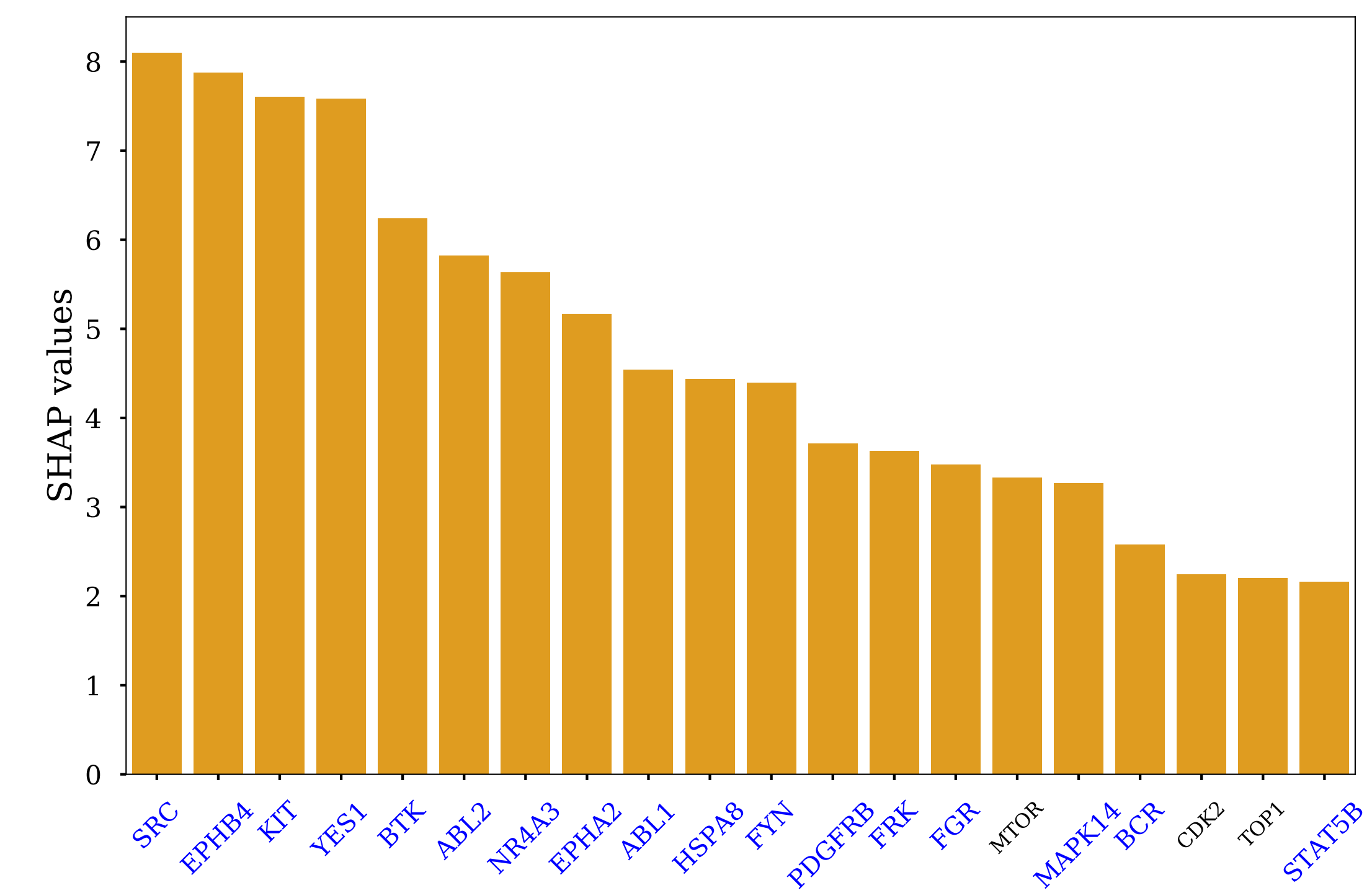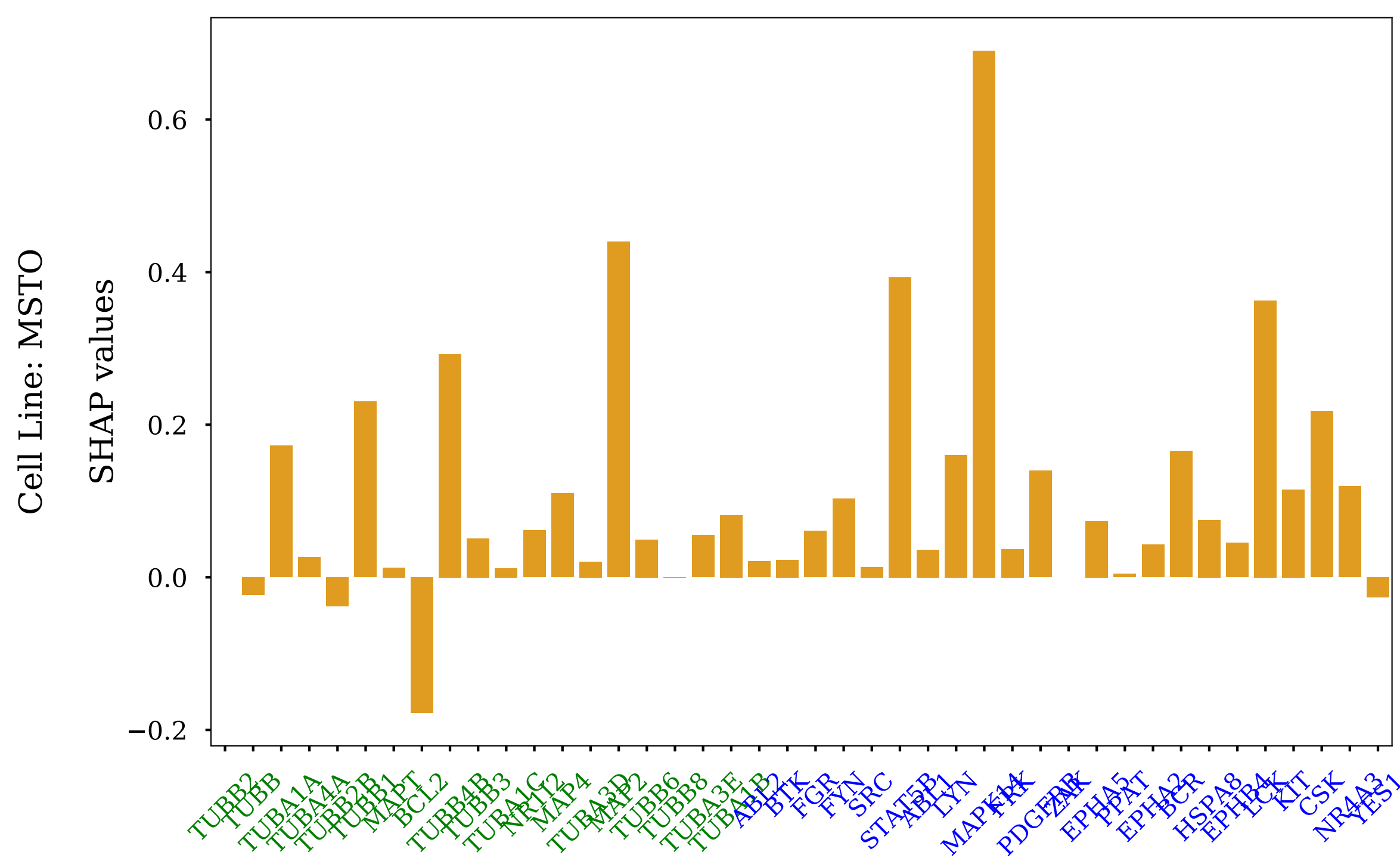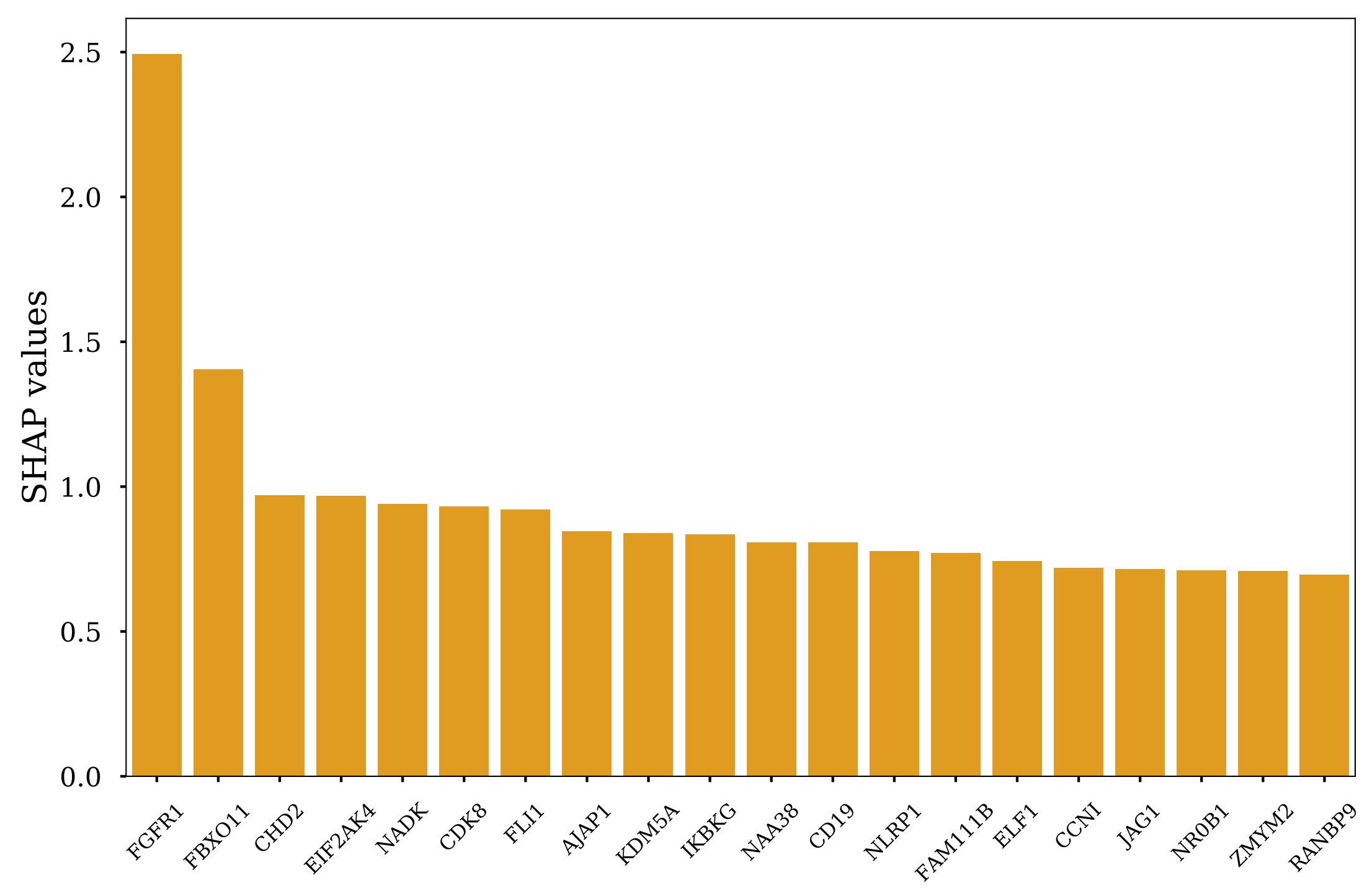

Supplement: S7 Fig — The left panel shows the SHAP values of drug targets, while the right panel shows the SHAP values of 20 genes with the most significant impact. (PDF) [file pcbi.1008653.s013.pdf]

Drug targets SHAP values

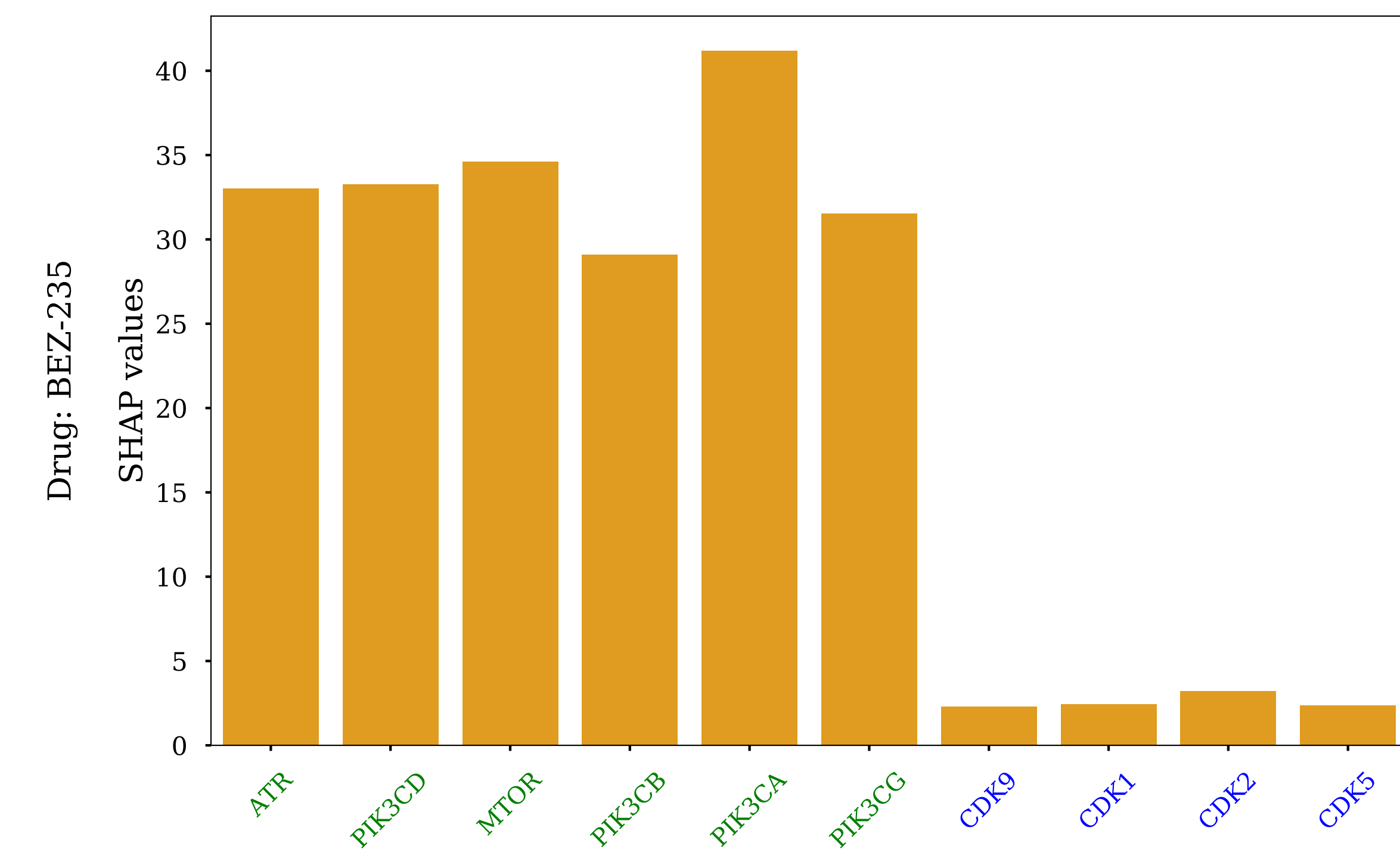

Top 20 genes with highest SHAP values

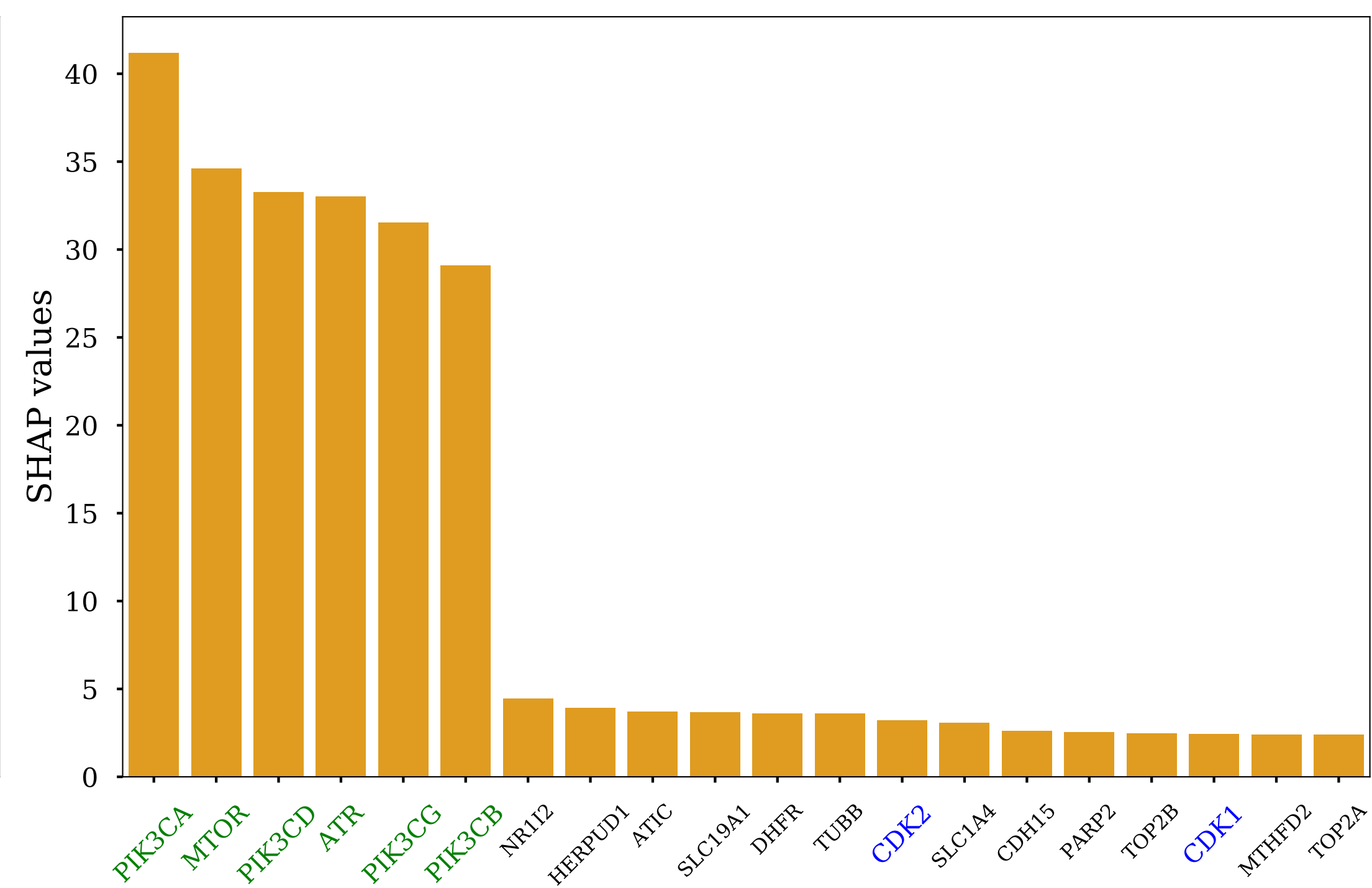

Drug: DINACICLIB

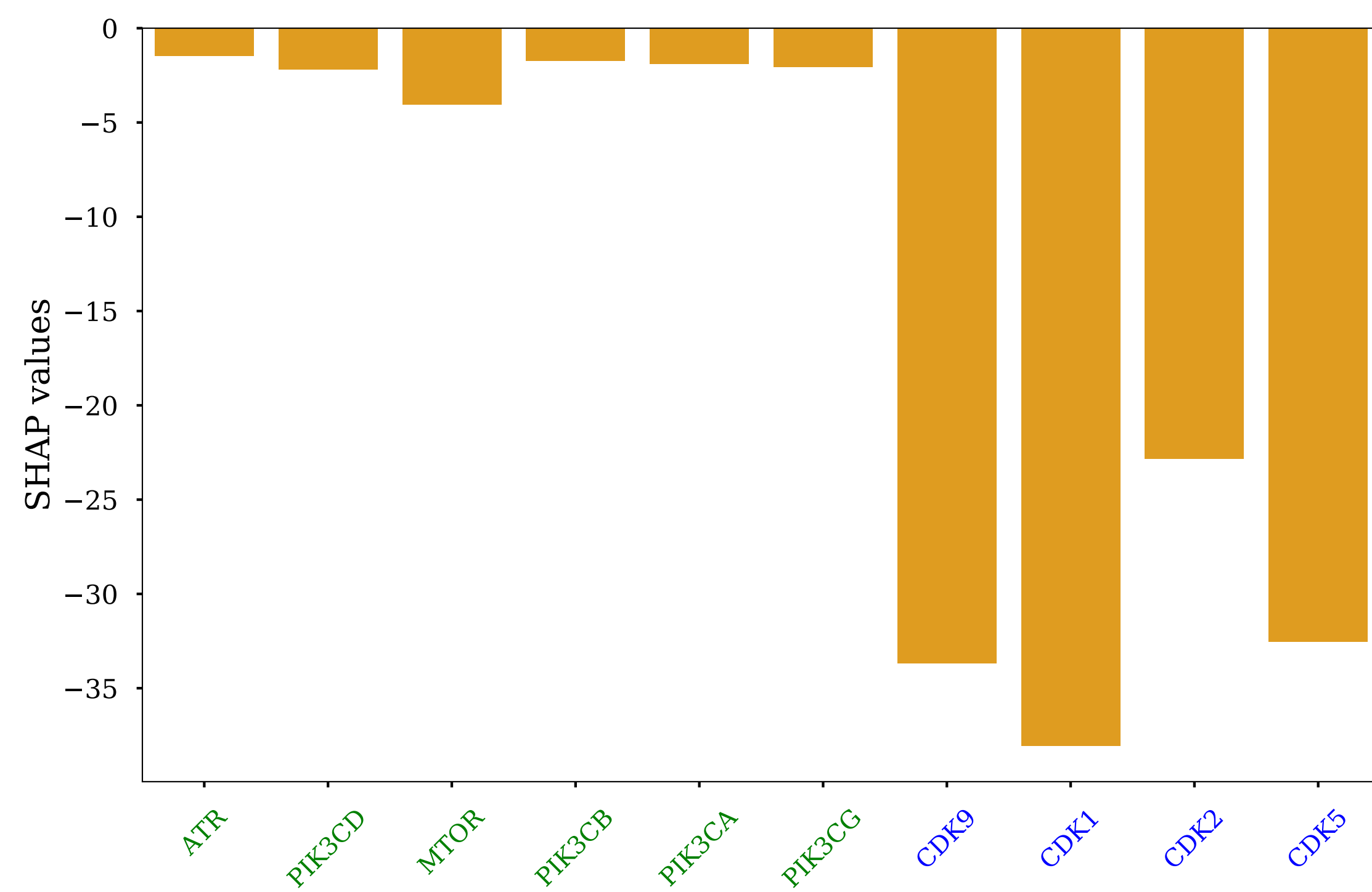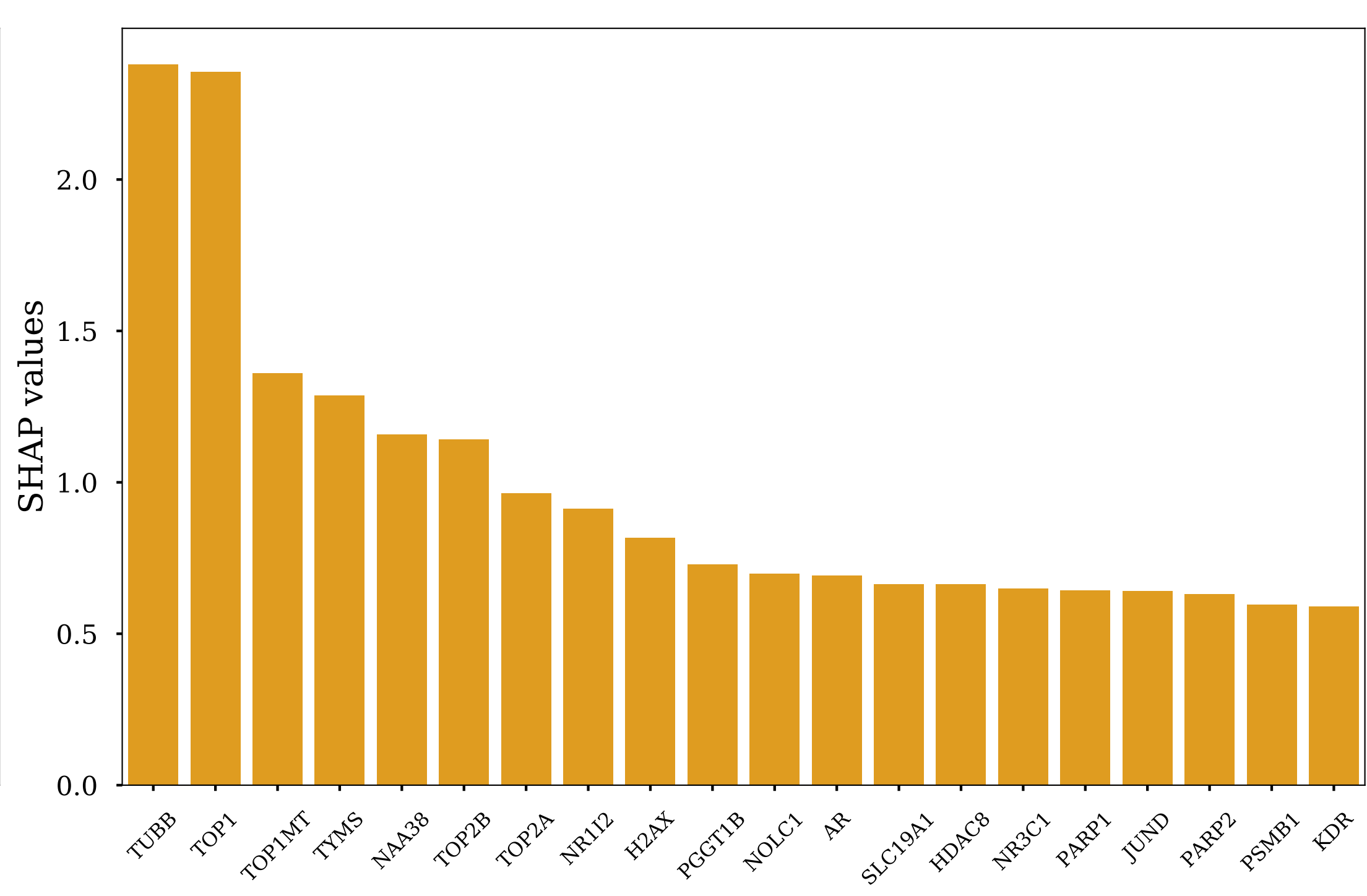

Cell Line: T47D

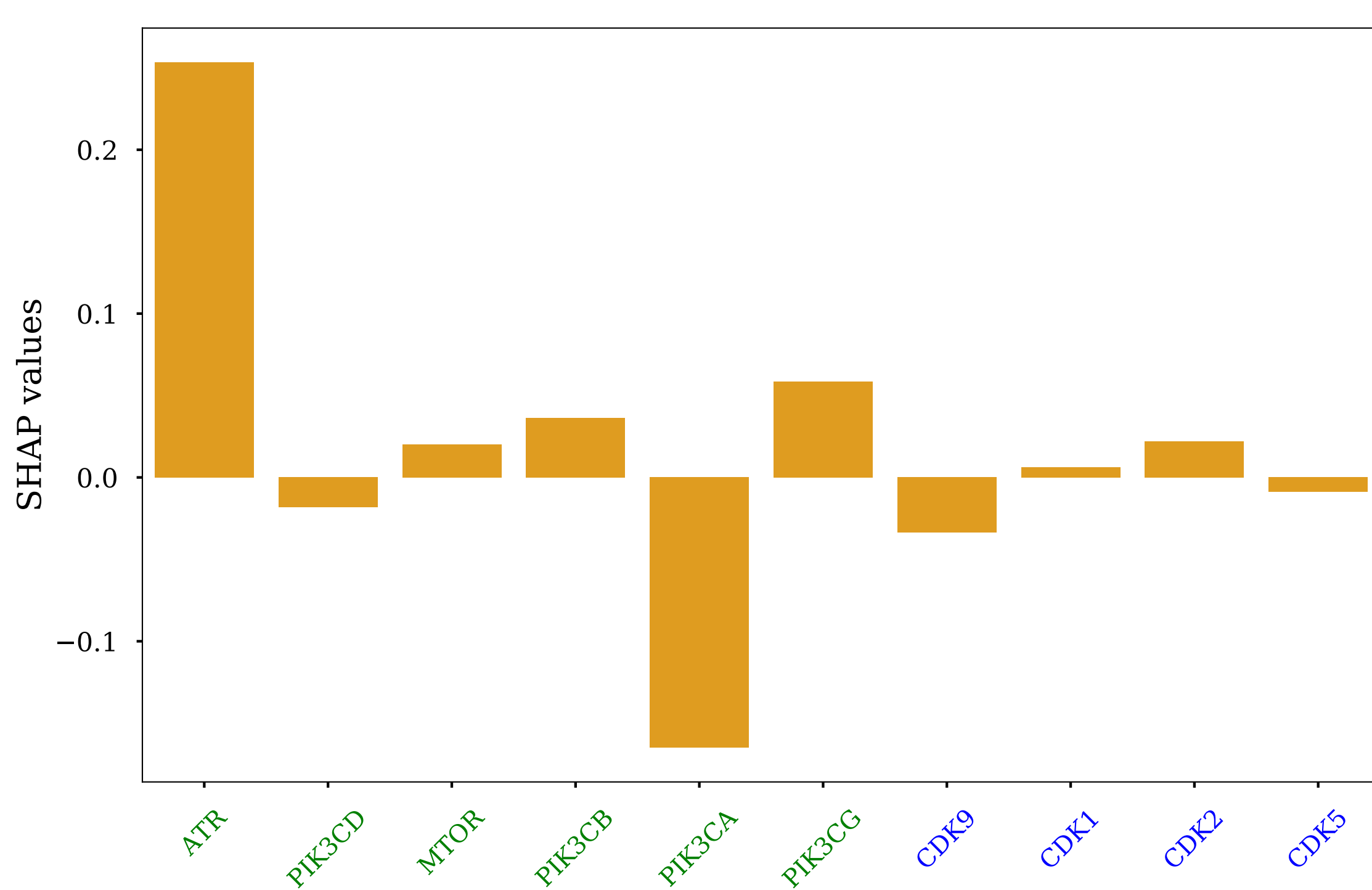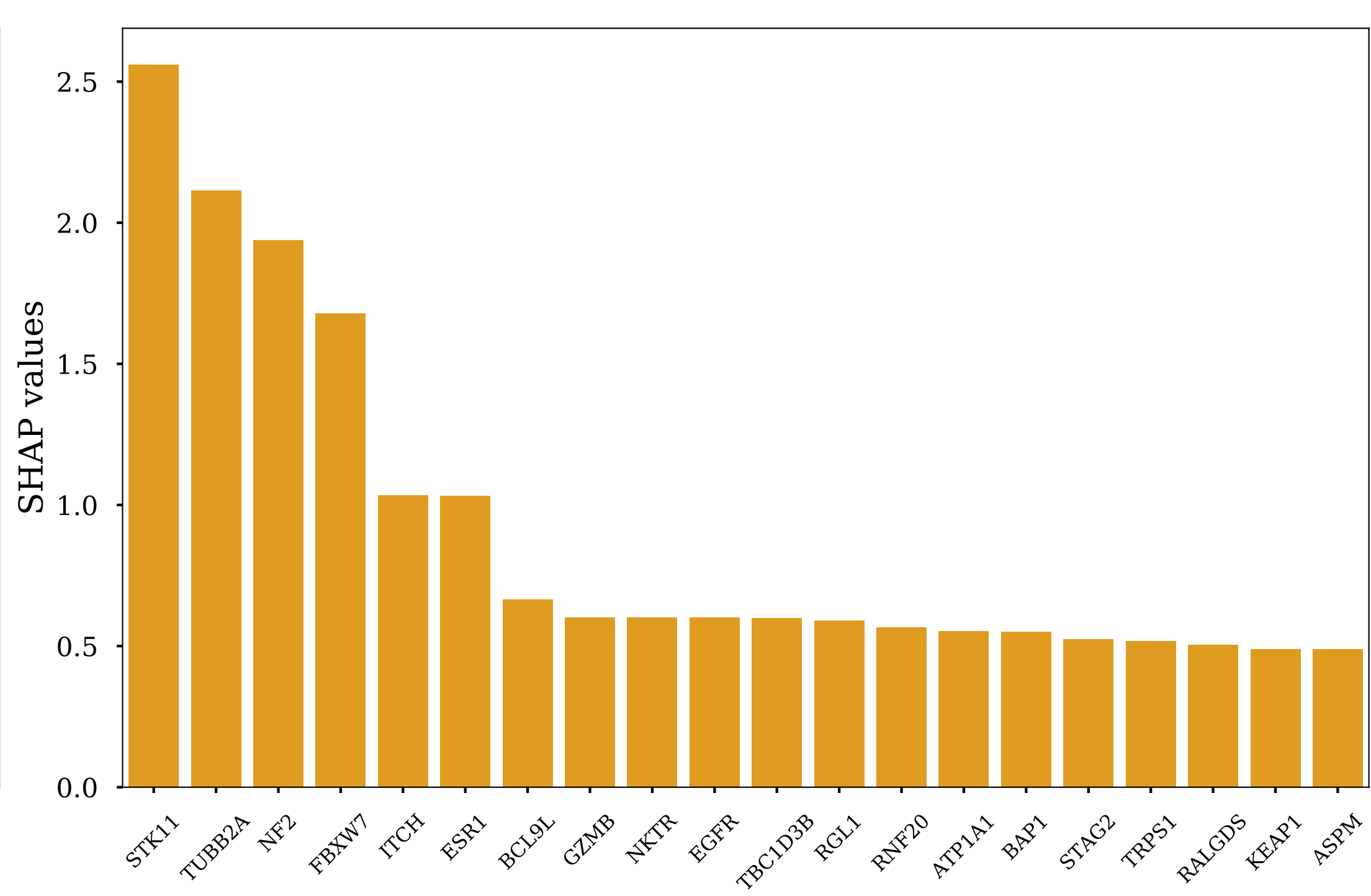

Supplement: S8 Fig — The left panel shows the SHAP values of drug targets, while the right panel shows the SHAP values of 20 genes with the most significant impact. (PDF) [file pcbi.1008653.s014.pdf]

Drug targets SHAP values

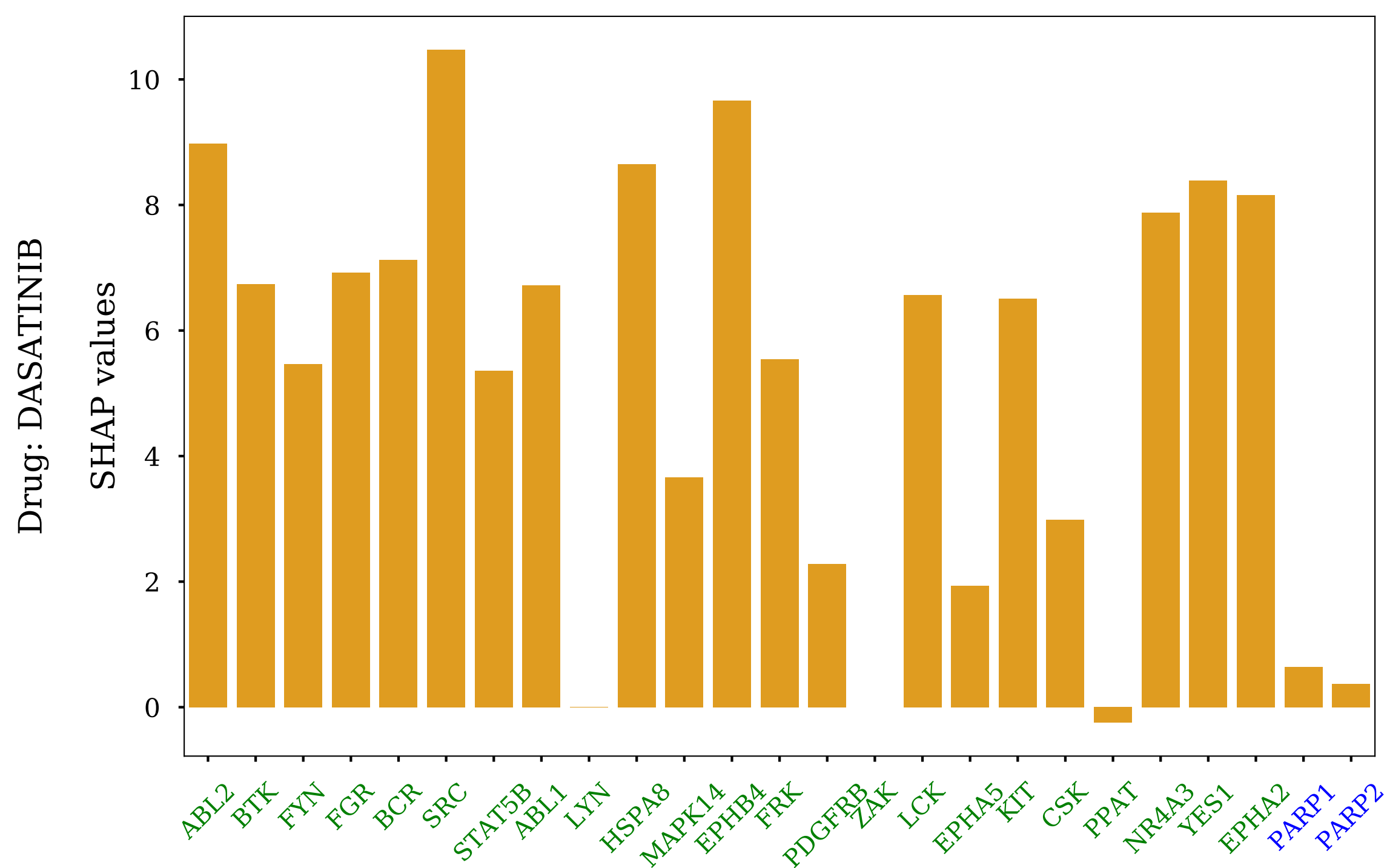

Top 20 genes with highest SHAP values

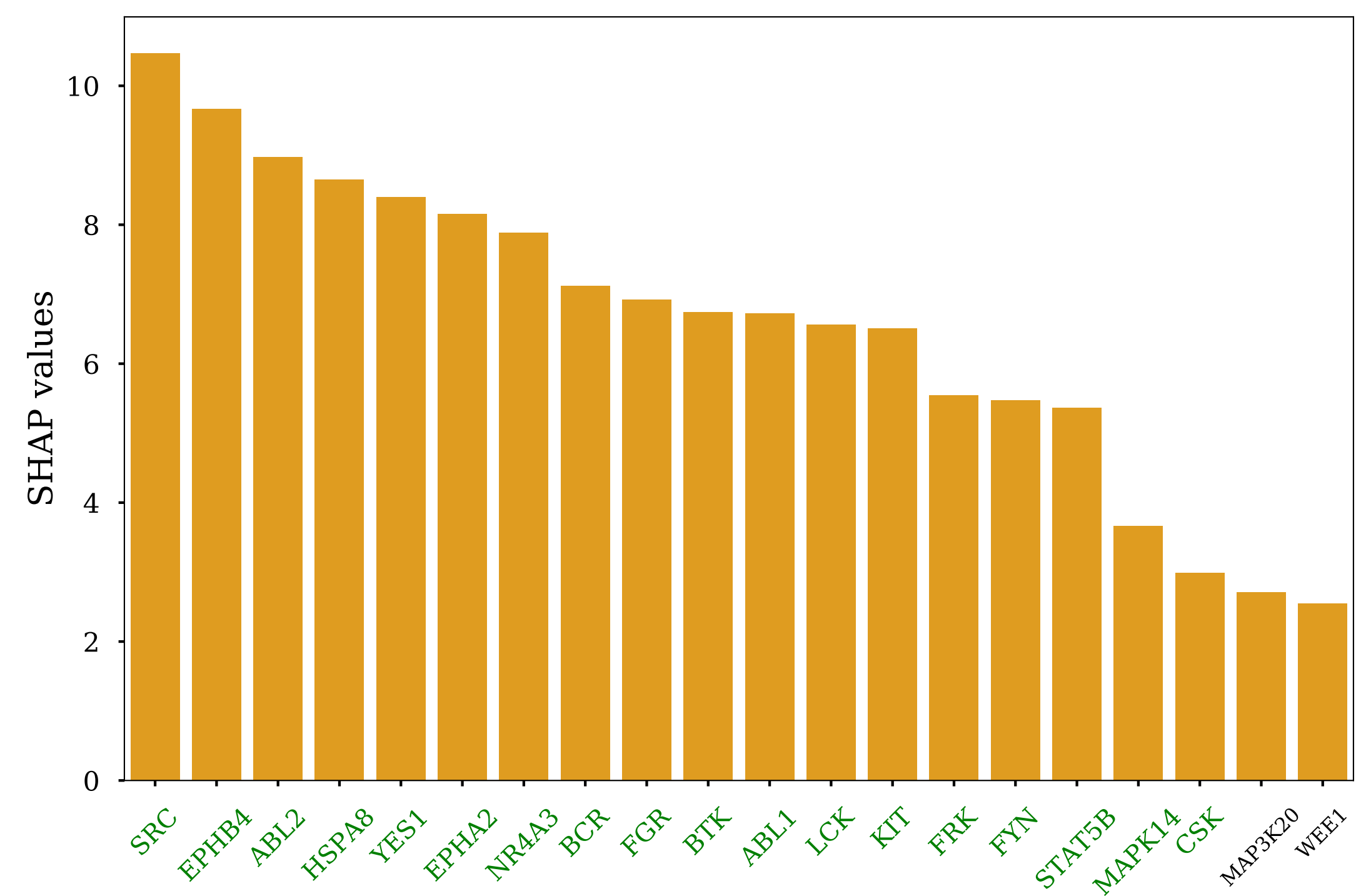

Drug: ABT-888

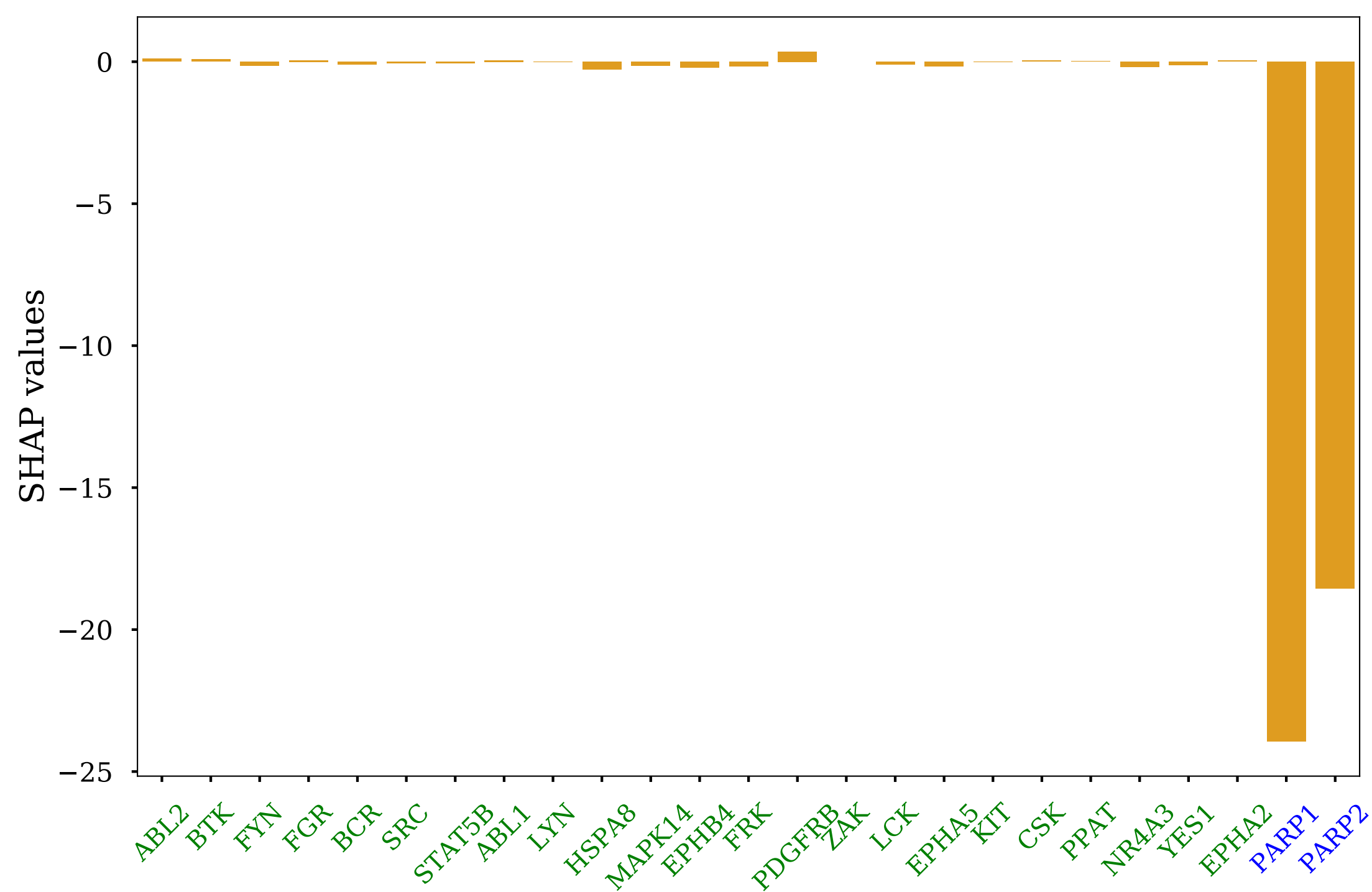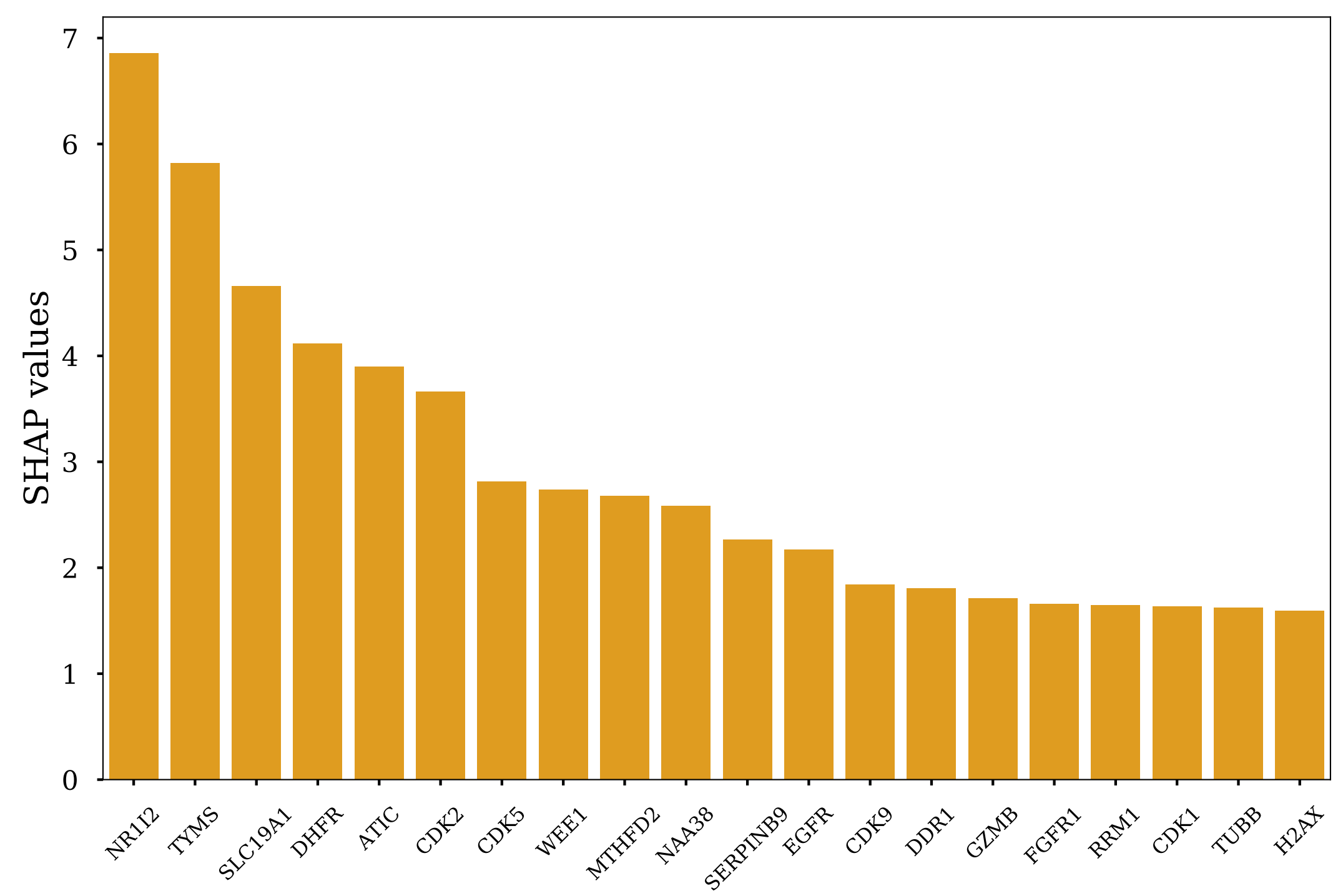

Cell Line: MSTO

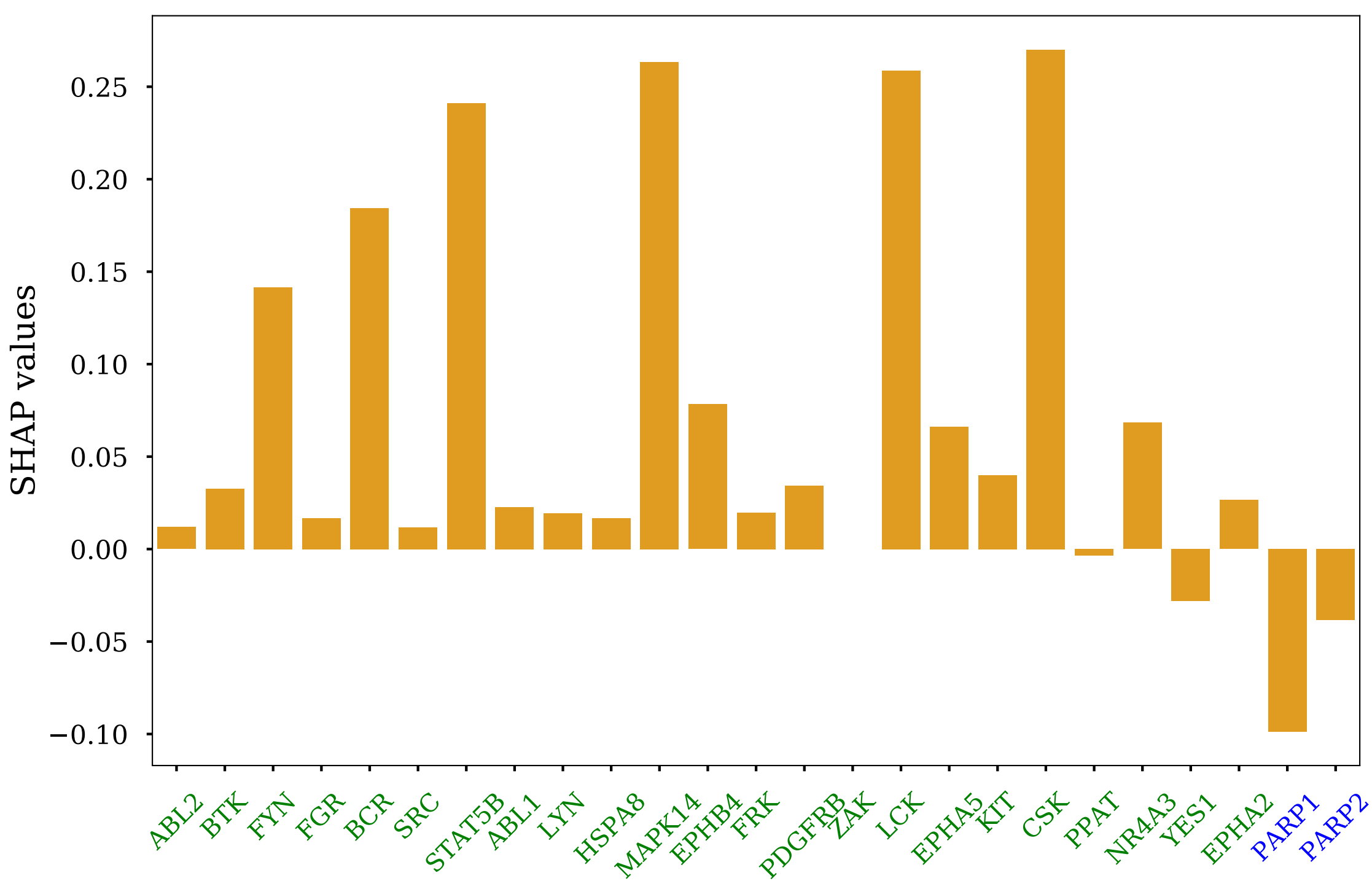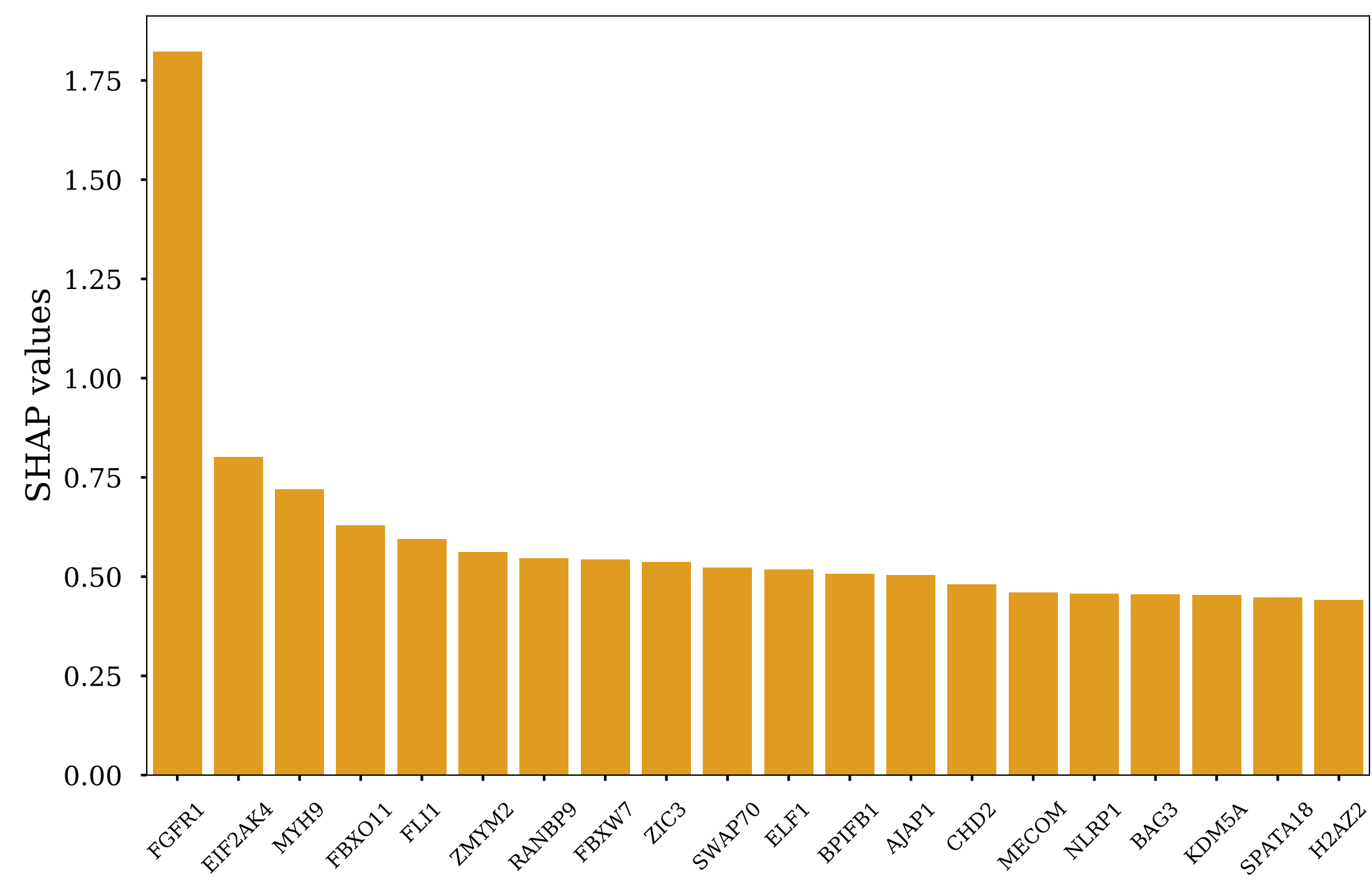

Supplement: S9 Fig — The left panel shows the SHAP values of drug targets, while the right panel shows the SHAP values of 20 genes with the most significant impact. (PDF) [file pcbi.1008653.s015.pdf]
