# Supplementary material for: TranSynergy: Mechanism-driven interpretable deep neural network for the synergistic prediction and pathway deconvolution of drug combinations
Source: PLoS Comput Biol. 2021 Feb 12;17(2):e1008653. doi: 10.1371/journal.pcbi.1008653 (PMC7906476; doi:10.1371/journal.pcbi.1008653)
Supplement: S2 Fig — Extra drug features, graphical neural fingerprint for drug A and drug B extracted with a GCN layer, were concatenated with the output of the Transformer and input into the last fully connected component. (PDF) [file pcbi.1008653.s008.pdf]

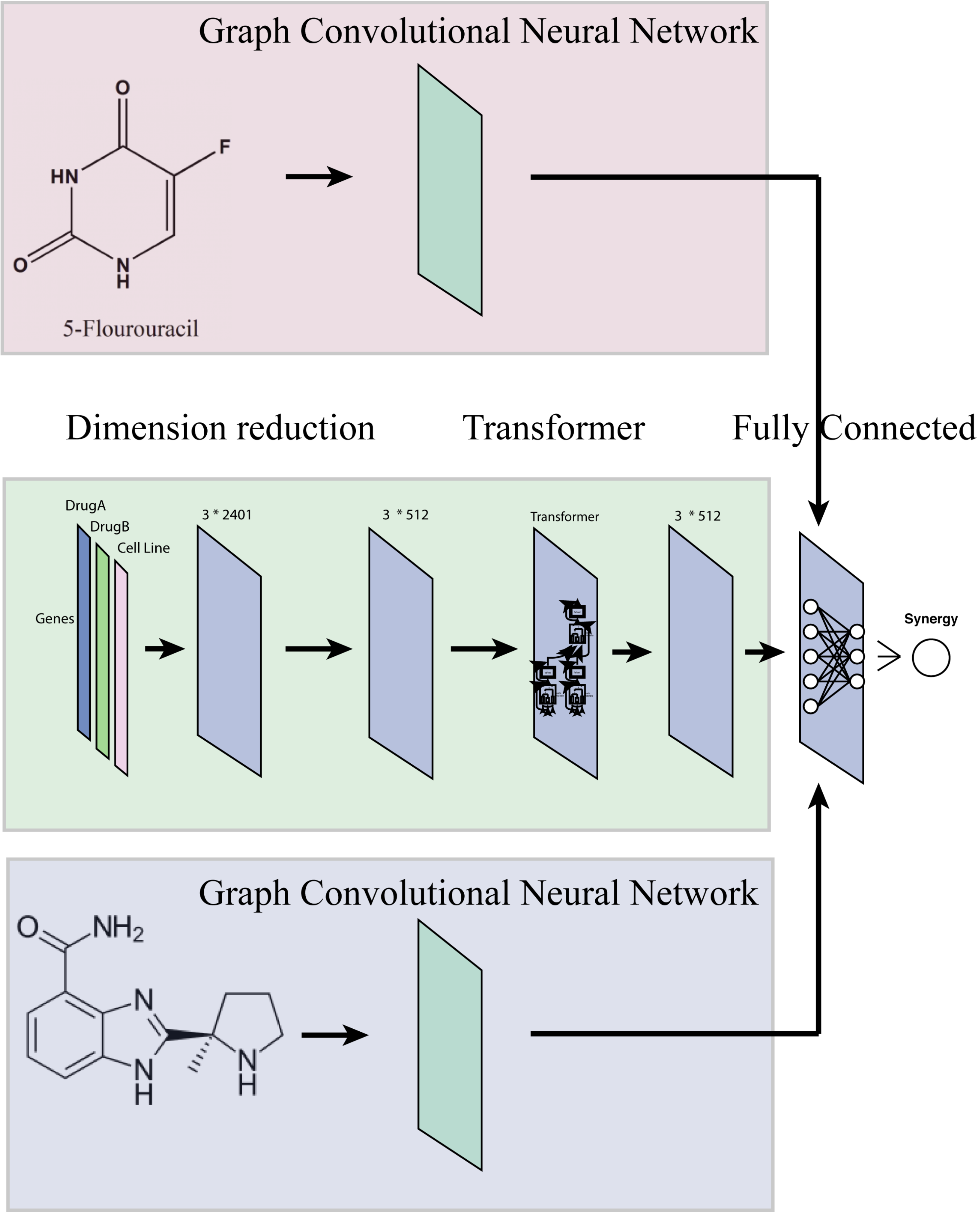

Graph Convolutional Neural Network
5-Flourouracil
Dimension reduction
Transformer
Fully Connected
DrugA
DrugB
Cell Line
Genes
3 * 2401
3 * 512
Transformer
3 * 512
Synergy
Graph Convolutional Neural Network
