# Supplementary material for: TranSynergy: Mechanism-driven interpretable deep neural network for the synergistic prediction and pathway deconvolution of drug combinations
Source: PLoS Comput Biol. 2021 Feb 12;17(2):e1008653. doi: 10.1371/journal.pcbi.1008653 (PMC7906476; doi:10.1371/journal.pcbi.1008653)
Supplement: Attachment — Submitted filename: ResponseToReviewers.pdf [file pcbi.1008653.s016.pdf]

Response:

We sincerely thank the reviewers for her/his constructive suggestions. The manuscript has been significantly modified to address the points raised by the reviewers. The details are summarized below and in the improved text of the manuscript. All revisions are highlighted red in the text.

Reviewer 1:

Major:

(1) Alpha is an important hyperparameter in RWR method. The authors show the impact of this parameter on the prediction results in Table S3 and use alpha = 0.5 in the experiments. Is this an 'overfitting'? The authors used the best results without tuning this hyperparameter to compare it to the other baseline methods. A detailed description is needed.

Thanks for the comments. To address the review's concern, we added the following to our manuscript to make the procedure clear: "To avoid information leaking and overfitting, we performed nested cross-validation for hyperparameter tuning and model selection. In each iteration, the data were split into training/validation/test sets. After the model was fitted using the training data set, optimal hyperparameter α in RWR was selected by the grid search over the validation set. The grid search determined the optimal α = 0.5 (S6 Table) over the validation set.".

S6 Table. Grid search results of the restart rates α, a hyperparameter in random walk with restart for network propagated drug target features inference, over the validation set.

Model	Restart rate (α)	Drug features	Cell line features	Spearman's Correlation	Pearson's Correlation
TranSynergy	0	Network propagated drug target profile	Gene dependencies	0.453±0.013	0.483±0.011
	0.1			0.712±0.014	0.719±0.013
	0.3			0.711±0.018	0.726±0.013
	0.5			**0.717±0.017**	**0.733±0.016**
	0.8			0.710±0.014	0.726±0.012

(2) The authors used RWR on a PPI network to represent the drug features instead of chemical information. It will be interesting to see the prediction results by integrating both drug features and chemical information in the TranSynergy model.

Thanks for the suggestion. To test the performance of combining chemical information, we twisted the current model and tested two methods to integrate chemical information as follows.

1. The new model integrated the physiochemical, toxophores, and fingerprint information of each drug, which is the same as that were used in DeepSynergy, as input (S1 Fig.). The difference between TranSyenrgy and TranSynergyCI is that the latter one concatenates this chemical information with

the output of the transformer and input into of last fully connected layer. The performance of this new model, TranSynergyCI, is slightly lower than the original TranSynergy model (S2 Table), suggesting the chemical structure does not add extra information. Technically, there are ~9000 extra features integrated into this new model, this causes the input dimension to dramatically increase. In turn, the complexity of the model and the number of trainable parameters in the model are increased. Secondly, A large amount of the drug's chemical information features are 0, so the input feature becomes sparse. Both of these two factors will introduce more possibilities for model fitting to stop in a local optimal and increase the difficulty to find the optimal parameters for this model. Fundamentally, the phenotypic response of drug actions come from a multi-scale process from drug-target interactions to their impact on a gene-gene interaction network. A drug-target interaction profile on a biological network can be more relevant to the drug-induced phenotype than a chemical structure.

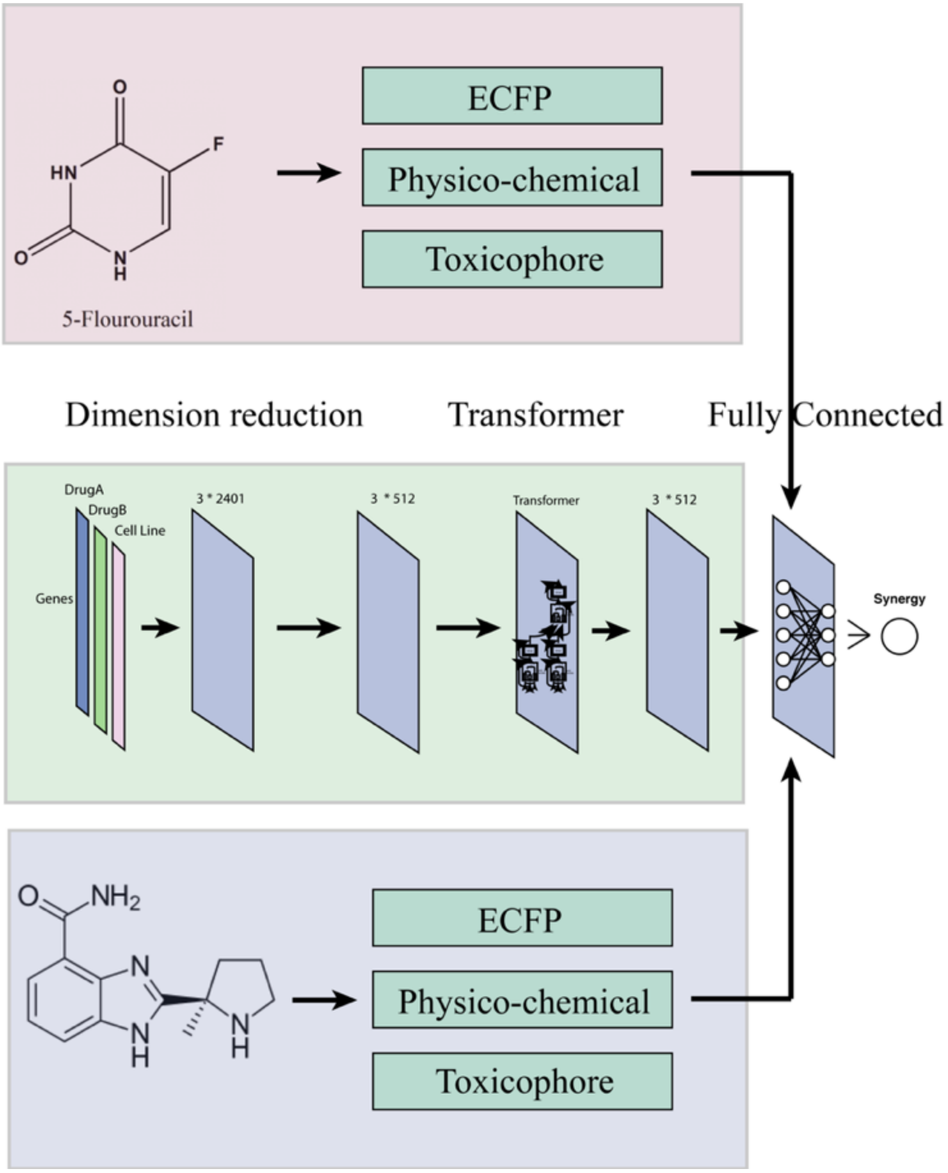

S1 Fig. The architecture of TranSynergy integrated with Chemical Information (TranSynergyCI). Extra drug features, including ECFP, physico-chemical and toxicophore features were concatenated with the output of the Transformer and input into the last fully connected component. These chemical features are the same as those used in deepSynergy.

S2 Table. Performance of TranSynergy integrated with Chemical Information (TranSynergyCI). Extra drug features, including ECFP, physico-chemical and toxicophore features were concatenated with the output of the Transformer and input into the last fully connected component. The architecture is shown in S1 Fig.

Model	Drug feature	Cell feature	Spearman correlation	Pearson correlation
TranSynergyCI	ECFP+phy+toxi + drug target	Gene expression	0.699 ± 0.009	0.711 ± 0.008
		Gene dependencies	0.712 ± 0.010	0.720 ± 0.011

2. In the second model, we integrated the drug features into TranSynergy by using a Graph Convolutional Neural Network (GCN) layer to extract drug graphic neural fingerprint (S2 Fig.). Two drug graphic neural fingerprints are concatenated with the output of the transformer and were used as the input of the last fully connected layer. The GCN layer could extract chemical information from drug molecular structures. Intuitively, the drugs were parsed as graph structures comprised of atoms that are inferred as the nodes of the graph, and bonds that are inferred as the edges of the graph. The output of this GCN layer is the drug’s graphical neural fingerprint. TranSynergyGNF's performance is still inferior to TranSynergy, which may be due to similar reasons as described before. (S3 Table).

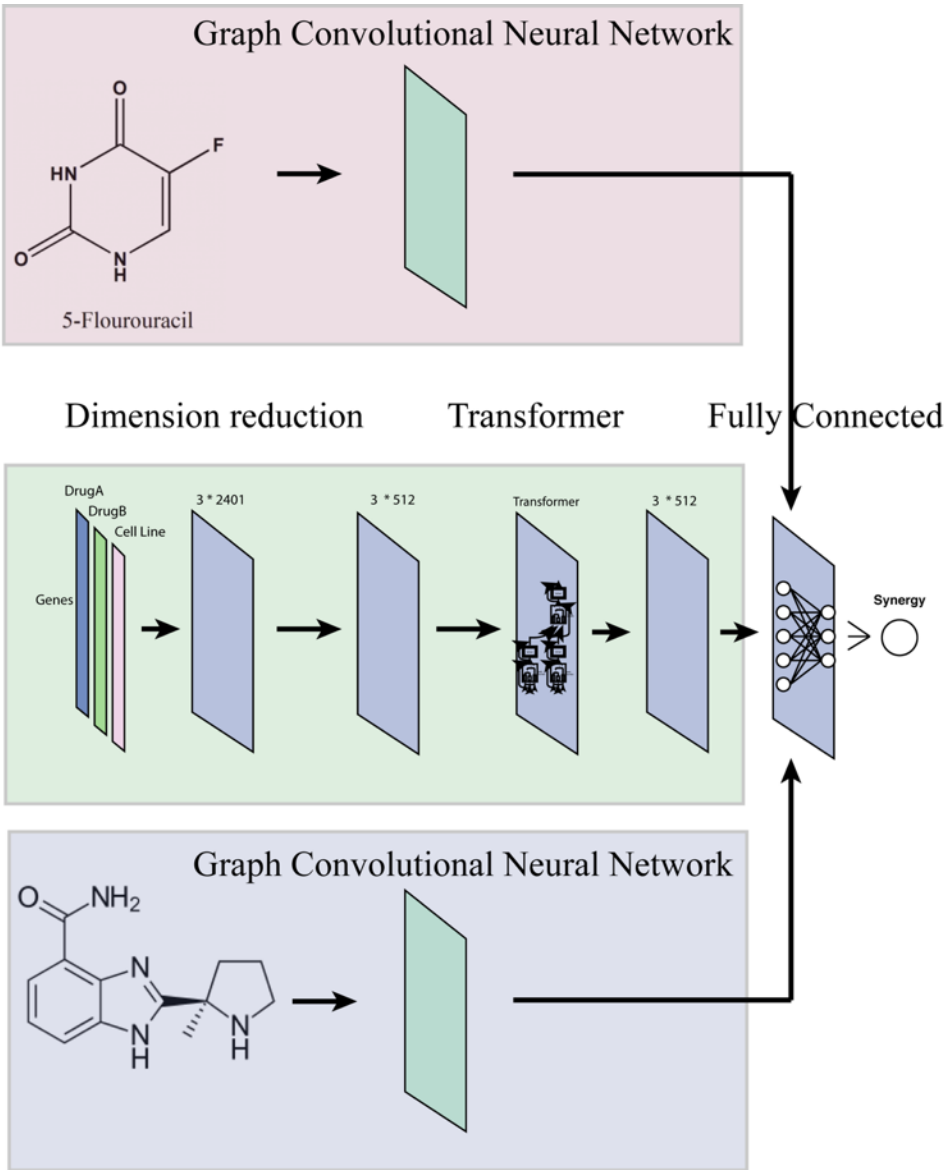

S2 Fig. The architecture of TranSynergy integrated with Graphical Neural Fingerprint (TranSynergyGNF). Extra drug features, graphical neural fingerprint for drug A and drug B extracted with a GCN layer, were concatenated with the output of the Transformer and input into the last fully connected component.

S3 Table. Performance of TranSynergy integrated with Graphical Neural Fingerprint (TranSynergyGNF). Extra drug features, graphical neural fingerprint for drug A and drug B extracted with a GCN layer, were concatenated with the output of the Transformer and input into the last fully connected component. The architecture is shown in S2 Fig.

Model	Drug feature	Cell line feature	Pearson	Spearman
TranSynergyGNF	Drug target + chemical structure	Gene dependecies	0.739±0.013	0.722±0.011

		Gene expression	0.730±0.015	0.716±0.022

Both new methods failed to boost model performance by integrating chemical information. This might indicate that adding extra chemical information didn't necessarily increase the prediction accuracy in this experimental setup. All the aforementioned results were collected in leave drug combination out scenario (Fig 3A).

(3) It is also better to show the baseline performance in Figure 3, since the improvement from TranSynergy can be cancer specific.

Thanks for the comments. To address the reviewer's concern, we added the cell line and tissue-specific performance from the baseline model too. It shows that TranSynergy outperforms DeepSynergy in five out of six tissues (Fig 4).

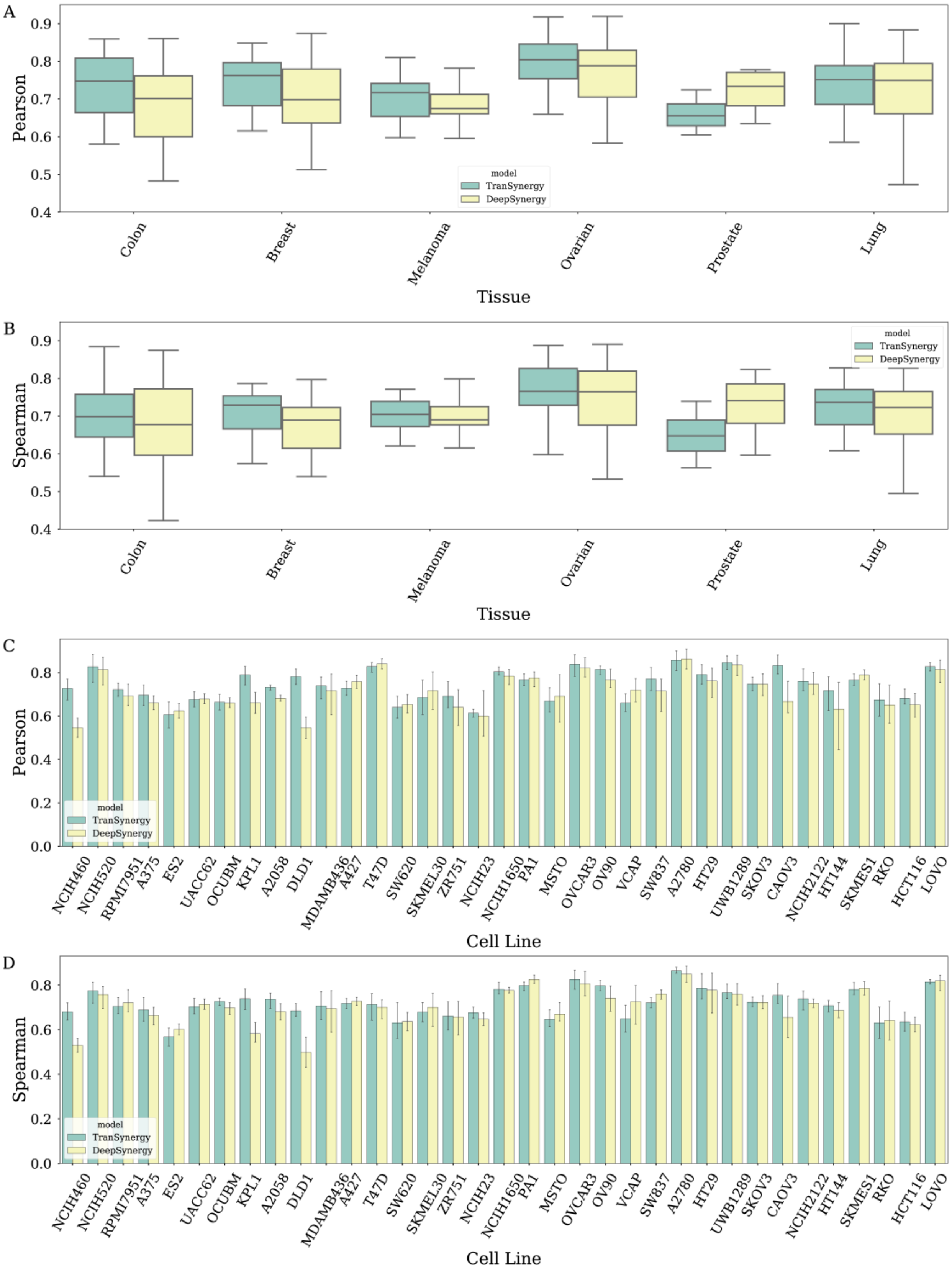
A
Pearson
Tissue
Colon
Breast
Melanoma
Ovarian
Prostate
Lung
model
TranSynergy
DeepSynergy
B
Spearman
Tissue
C
Pearson
Cell Line
NCIH460
NCIH520
RPMI7951
A375
ES2
UACC62
OCUBM
KPL1
A2058
DLD1
MDAMB436
A427
T47D
SW620
SKMEL30
ZR751
NCIH23
NCIH1650
PA1
MSTO
OVCAR3
OV90
VCAP
SW837
A2780
HT29
UWB1289
SKOV3
CAOV3
NCIH2122
HT144
SKMES1
RKO
HCT116
LOVO
D
Spearman
Cell Line

Fig 4. Tissue-specific and cell line-specific prediction performances of TranSynergy and DeepSynergy. The top two panels (A and B) are tissue-specific performance with Pearson correlation and Spearman correlation as the metrics. The bottom two panels (C and D) are cell line-specific performance with Pearson correlation and Spearman correlation as the metrics.

Minor:

(1) It is better to show that whether the improvement by TranSynergy in Table 1 and Table 2 are statistical significance.
Thanks for the suggestions. We added in the statistical significance in Table 1, Table 2, Table 3 and Table 4.

Reviewer 2:

In terms of performance, the authors claim that TranSynergy along with certain features can outperform deepsynergy. However there are some issues, first the main text does not include the number of cross validation steps the authors performed. Second, deepsynergy includes several other benchmarks other than correlation coefficients, such as mean squared error, ROC AUC, precision, recall, etc. that the authors do not include in this analysis. Because of this, their approach is not an apples-to-apples comparison, as the authors claim. This issue is persistent through to figure 3 and table 4, which only includes correlation coefficients and does not include other metrics of classifier performance.

Thanks for the comments. We added the following part to the current version of manuscript to clarify our experimental procedures and to address the reviewer's concern.

"Model evaluations were performed in three scenarios. a) In the leave drug combination out scenario, we applied the nested cross-validation method. The data was split into five folds such that the drug pair in one fold does not overlap with the drug pairs in other folds (Fig 3A). Because the drug combination of drug A with drug B and drug B with drug A should have the same drug combination synergy score, the size of training data was doubled by swapping the drug A and drug B. The model was iteratively trained/validated in four folds and then tested in the remaining one fold. b) We further tested our models in the most challenging leave cell out scenario. We used affinity propagation methods to group the cell lines into 4 clusters based on cell line features similarity (Fig 3B). Since 28 cell lines are in the biggest cluster, we used them as the training/validation dataset and held out the remaining cell lines in other clusters as the test dataset (Fig 5). In this way, the cell lines in the testing set are significantly different from those in the training/validation set. c) In the leave drug out scenario, we grouped the drugs into 5 clusters with the affinity propagation method based on their structural similarities (Fig 3C and 6). We chose drugs in one cluster and held out all drug pairs including these three drugs as the test set. The remaining drug pairs are the training/validation set. We performed three tests with keeping out the drugs in each of the three smallest clusters. In each test, the ratio of samples in the training and the testing data is around 4:1."

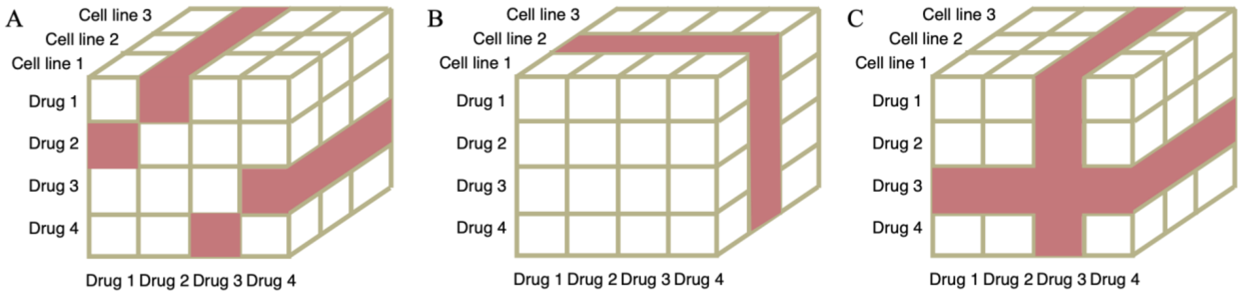

Fig 3. Illustration of three training/test dataset splitting scenarios. Each data point is composed of two drugs and a cell line. The data samples in white and red are the training/validation dataset and test dataset, respectively. A) Leave drug combination out scenario. In this example, drug pairs, including drug1+drug2 and drug_3+drug4, are colored red and held out as test dataset. B) Leave cell lines out scenario. In this example, the cell line 2 is colored red and held out as test dataset. C) Leave drugs out scenario. In this example, the drug 3 is colored red and held out as test dataset.

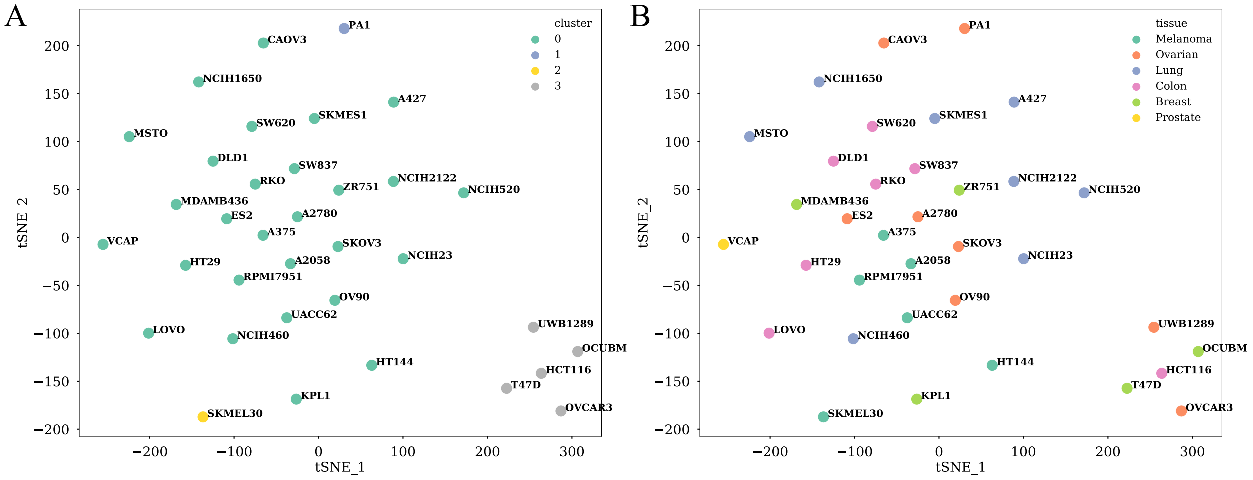

Fig 5. Visualization of different cell lines with t-SNE analysis. High dimensional cell lines vector representations are projected into 2d space with the first two t-SNE components. A) Different colors indicate assigned clusters of each cell line by the affinity propagation method. B) Different colors indicate different tissues of each cell line.

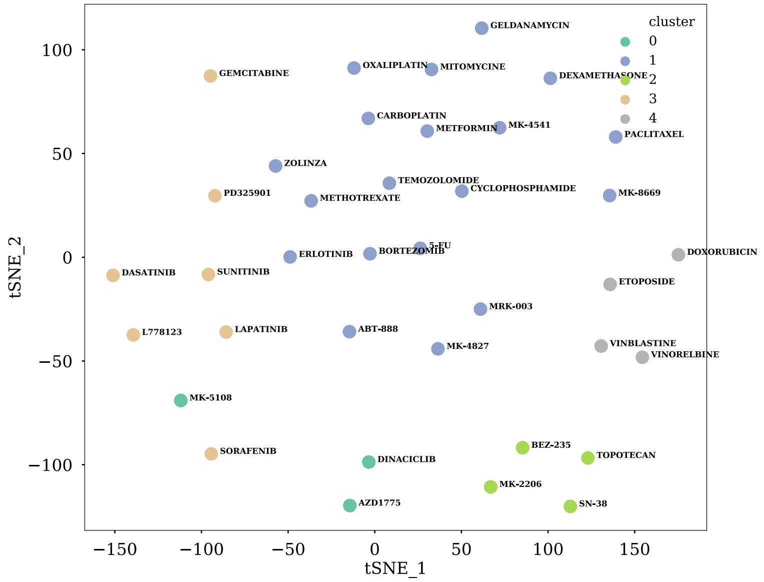

Fig 6. Visualization of drug features with t-SNE analysis. High dimensional cell lines vector representations are projected into 2d space with the first two t-SNE components. Different colors indicate assigned clusters of each drug by affinity propagation method.

2) We added other metrics including mean square error, ROC-AUC, and PR-AUC. ROC-AUC and PR-AUC are used to show the classifier performance (Table 2 and 4). As mentioned in the DeepSynergy paper, the precision and recall score requires an appropriately selected threshold for the predicted values to determine whether a sample is a positive sample or a negative sample. The PR-AUC which combines the information for precision and recall does not require the selection of the threshold. Thus, we used these PR-AUC and ROC-AUC to show the classifier's performance.

Table 2. Performance comparison of TranSynergy with different cell line features to DeepSynergy model in classification scenarios. *TranSynergy that uses gene expression+Gene dependency cell line features was used to compute the statistical significance of performance difference between TranSynergy and DeepSynergy.

Models	Drug features	Cell line features	PR-AUC	ROC-AUC
TranSynergy	Network propagated drug target profile	Gene dependency	0.625±0.020	**0.908±0.011**
		Gene expression	0.618±0.016	0.901±0.009
		Gene expression + Gene dependency	**0.627±0.013**	0.907±0.011

DeepSynergy	Physicochemical properties, toxicophores and fingerprints	Gene expression	0.594±0.021	0.900±0.009
Statistical significance* (p-value)			<0.0001	0.0134

Table 4. Performance of TranSynergy and DeepSyenrgy on leave cell out scenario and leave drug out scenario for the classification task.

Model	Leave cells out		Leave drugs out	
	ROC-AUC	PR-AUC	ROC-AUC	PR-AUC
TranSynergy	**0.808 ± 0.017**	**0.372 ± 0.022**	**0.778 ± 0.032**	**0.406 ± 0.106**
DeepSynergy	0.805 ± 0.017	0.360 ± 0.022	0.753 ± 0.030	0.378 ± 0.075
Statistical significance (p-value)	0.3328	0.0908	0.0034	0.1379

Next the authors attempt to describe the cell line specific performance of their approach in figure 3. It is unclear in the main text the approach they used to predict cell-line specific performance. Did they train transsynergy on all other cell line data and test on a new cell line, or did they take 80% of all cell line data, and they are showing the performance on the hold out set? If it's the latter, then confidence intervals should be shown. It would also be important to show the number of samples available for each cell line. Perhaps the low performance on prostate tissues may be due to low training data available for prostate cells, or the model is overfit to other cell lines.

Thanks for the comments.

1) We split data into five folds and performed nested cross-validation as described in the previous question. Therefore, the cell line-wise performance was evaluated in each fold, when they were used as the test dataset. The detailed procedure was added to the manuscript. We also added error bars to our cell line-wise performance plot (Fig 4) as suggested by the reviewer.

2) We showed the number of samples for each cell line in S4 Table. All cell lines have almost the same number of samples in the training dataset. Thus the number of samples for VCAP, which is the only cell line in prostate tissue, is the same as others. To visualize cell line differences, we also performed the t-SNE analysis to plot the cell line's high dimensional vector representation into a 2D space (Fig 5).

"The prostate tissue had the lowest Pearson correlation coefficient among all tissues included in our data. VCAP, which is the only prostate cancer cell line, had the third-lowest Pearson's correlation coefficient and Spearman's correlation coefficient across all cell lines. The mediocre performance of the VCAP cell line could be due to that it is not closely similar to other cell lines. To reflect relationships between cell lines, we performed the t-SNE analysis to visualize the high

dimensional vector representation of cell lines mapped to a 2D space with the first two t-SNE components (Fig 5). It showed that the VCAP cell line is located on the edge of one cluster, which indicates the VCAP cell line might have distinctive features. We also noticed that five cell lines were isolated from the remaining cell lines in the t-SNE plot. They included T47D and OCUBM from the breast tissue, HCT116 from the colon tissue, as well as UWB1289 and OVCAR3 from ovarian tissue. The variances of these three tissues were higher than those of the other three tissues. These larger variances among cell line features within these three tissues might contribute to the observed larger performance variances.

We additionally tested our models in the most challenging leave cells out scenario (Fig 3B). We used affinity propagation methods to divide the cell lines into 4 clusters based on cell line features. Since 28 cell lines are in the biggest cluster, we used them as the training dataset and held out the remaining cell lines as the test dataset. In this way, the cell lines in the test dataset are the most different from those in the training dataset. All the cell lines have a similar amount of data (S4 Table). This is a robust method to test the generalization power of the models. As shown in Table 3 and 4, the performance of the TranSynergy is slightly better than DeepSynergy but both are lower than the first scenario.”

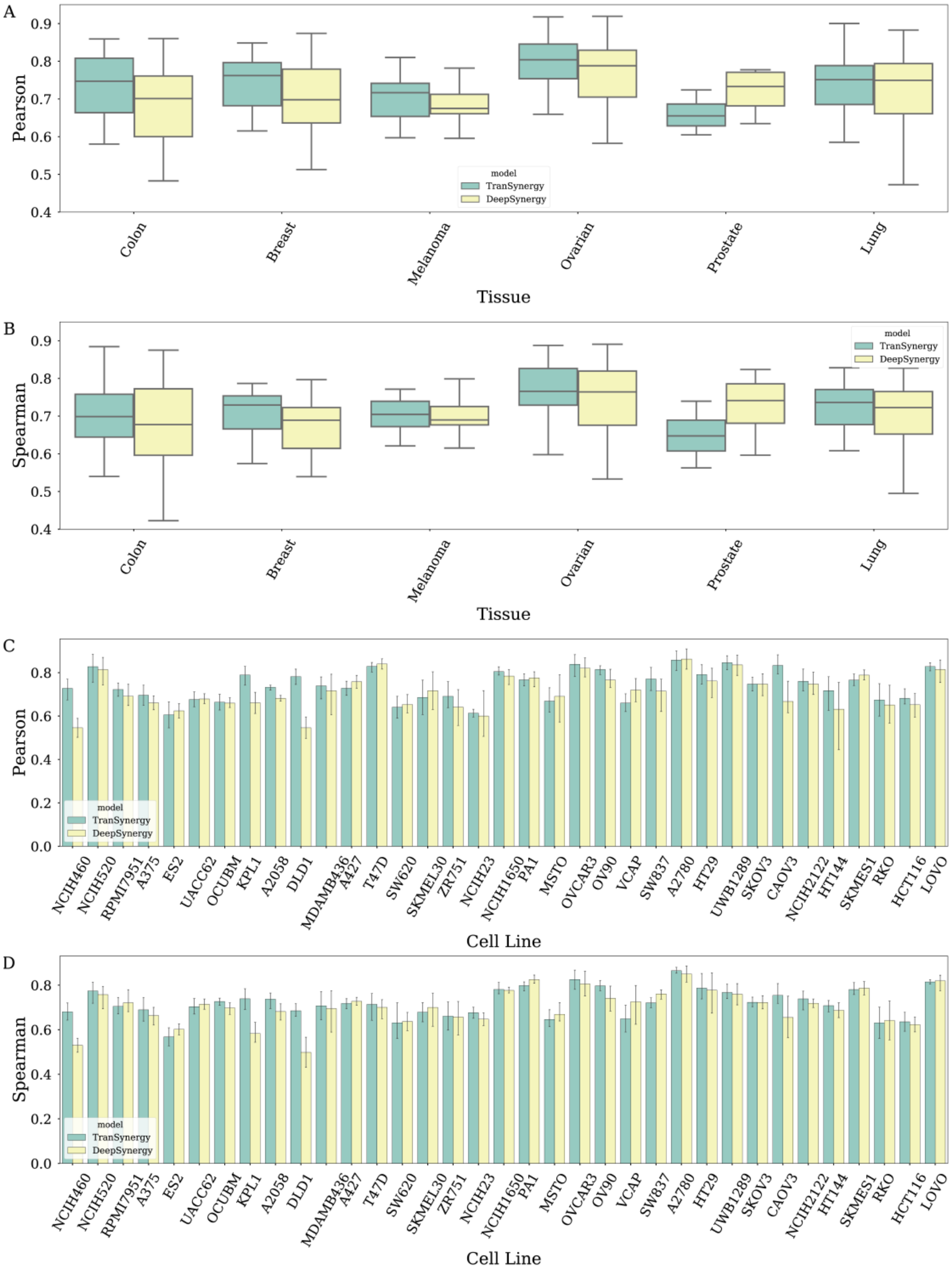

A
Pearson
Tissue
Colon
Breast
Melanoma
Ovarian
Prostate
Lung
model
TranSynergy
DeepSynergy
B
Spearman
Tissue
Colon
Breast
Melanoma
Ovarian
Prostate
Lung
model
TranSynergy
DeepSynergy
C
Pearson
Cell Line
NCIH460
NCIH520
RPMI7951
A375
ES2
UACC62
OCUBM
KPL1
A2058
DLD1
MDAMB436
A427
T47D
SW620
SKMEL30
ZR751
NCIH23
NCIH1650
PA1
MSTO
OVCAR3
OV90
VCAP
SW837
A2780
HT29
UWB1289
SKOV3
CAOV3
NCIH2122
HT144
SKMES1
RKO
HCT116
LOVO
model
TranSynergy
DeepSynergy
D
Spearman
Cell Line
NCIH460
NCIH520
RPMI7951
A375
ES2
UACC62
OCUBM
KPL1
A2058
DLD1
MDAMB436
A427
T47D
SW620
SKMEL30
ZR751
NCIH23
NCIH1650
PA1
MSTO
OVCAR3
OV90
VCAP
SW837
A2780
HT29
UWB1289
SKOV3
CAOV3
NCIH2122
HT144
SKMES1
RKO
HCT116
LOVO
model
TranSynergy
DeepSynergy

Fig 4. Tissue-specific and cell line-specific prediction performances of TranSynergy and DeepSynergy. The top two panels (A and B) are tissue-specific performance with Pearson correlation and Spearman correlation as the metrics. The bottom two panels (C and D) are cell line-specific performance with Pearson correlation and Spearman correlation as the metrics.

Finally the authors show that using their variant of GSEA can identify pathways involved in the PPI of drugs. What would be helpful here would be a comparison of their shapely method and typical GSEA results. And observing the differences between the top pathways associated with either.

Thanks for the suggestions. Typical GSEA requires the differential gene expression profiles between cell lines that are treated by a drug combination and those untreated. Unfortunately, such data are not available for the cases tested in this manuscript. We have added this discussion in the revision.

Other general comments:
While the author's approach for representing drugs as networks of their PPI is interesting, there might be important chemical properties that the authors might be missing by not incorporating chemical features.

Thanks for the suggestion. To test the necessities of integrating chemical information, we twisted current model and tested two methods to integrate chemical information as follows.

1. The new model integrated the physiochemical, toxophores, and fingerprint information of each drug, which is the same as that were used in DeepSynergy, as input (S1 Fig., next page). The difference between TranSyenrgy and TranSynergyCI is that the latter one concatenates this chemical information with the output of the transformer and input into of last fully connected layer. The performance of this new model, TranSynergyCI, is slightly lower than the original TranSynergy model (S2 Table), suggesting the chemical structure does not add extra information. Technically, there are ~9000 extra features integrated into this new model, this causes the input dimension to dramatically increase. In turn, the complexity of the model and the number of trainable parameters in the model are increased. Secondly, A large amount of the drug's chemical information features are 0, so the input feature becomes sparse. Both of these two factors will introduce more possibilities for model fitting to stop in a local optimal and increase the difficulty to find the optimal parameters for this model. Fundamentally, the phenotypic response of drug actions come from a multi-scale process from drug-target interactions to their impact on a gene-gene interaction network. A drug-target interaction profile on a biological network can be more relevant to the drug-induced phenotype than a chemical structure.

S2 Table. Performance of TranSynergy integrated with Chemical Information (TranSynergyCI). Extra drug features, including ECFP, physico-chemical and toxicophore features were concatenated with the output of the Transformer and input into the last fully connected component. The architecture is shown in S1 Fig.

Model	Drug feature	Cell feature	Spearman correlation	Pearson correlation

TranSynergyCI	ECFP+phy+toxi + drug target	Gene expression	0.699 ± 0.009	0.711 ± 0.008
		Gene dependencies	0.712 ± 0.010	0.720 ± 0.011

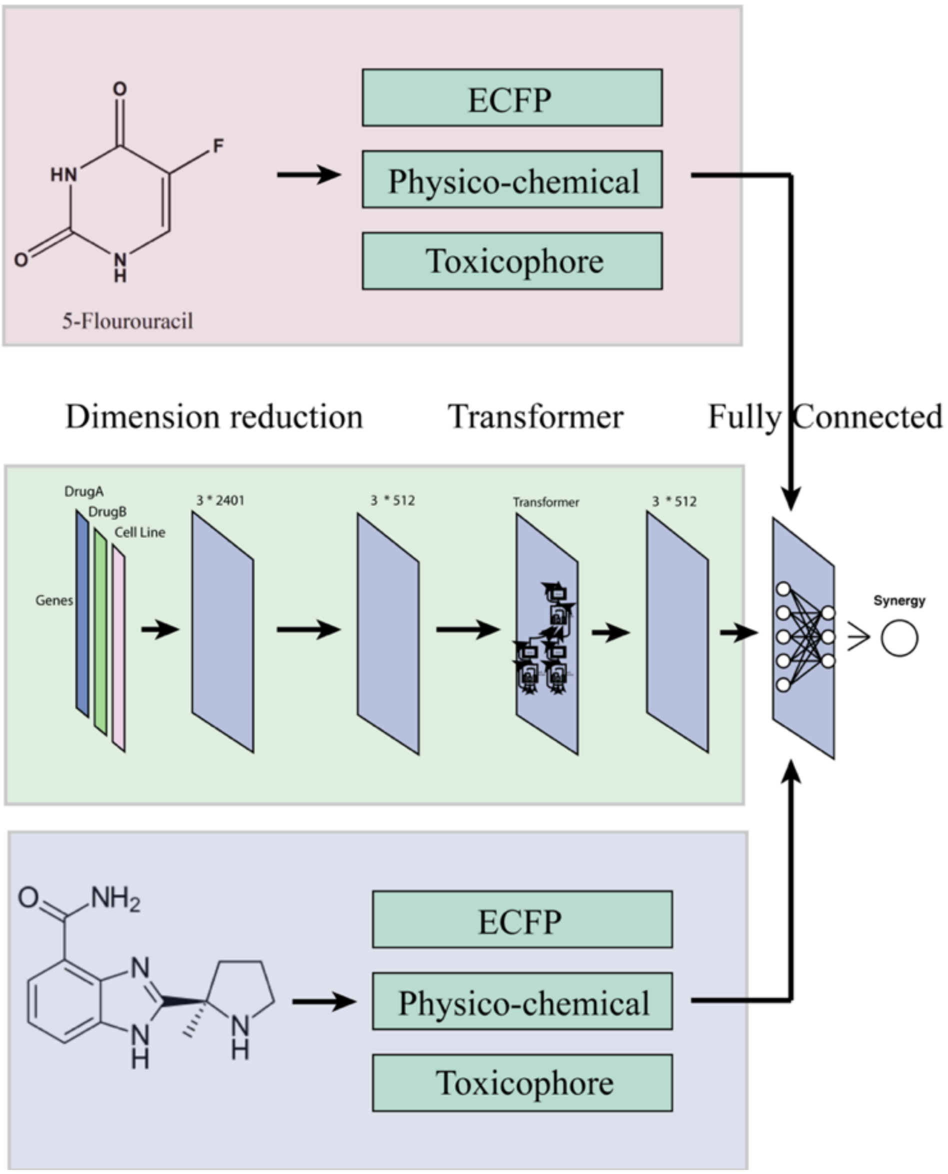

S1 Fig. The architecture of TranSynergy integrated with Chemical Information (TranSynergyCI). Extra drug features, including ECFP, physico-chemical and toxicophore features were concatenated with the output of the Transformer and input into the last fully connected component. These chemical features are the same as those used in deepSynergy.

2. In the second model, we integrated the drug features into it by using a Graph Convolutional Neural Network (GCN) layer to extract drug graphic neural fingerprint (S2 Fig.). Two drug graphic neural fingerprints are concatenated with the output of the transformer and were used as the input of the last fully connected layer. The GCN layer could extract chemical information from drug molecular

structures. Intuitively, the drugs were parsed as graph structures comprised of atoms, which are inferred as the nodes of the graph, and bonds, which are inferred as the edges of the graph. The output of this GCN layer is the drug's graphical neural fingerprint. TranSynergyGNF's performance is still inferior to TranSynergy (S3 Table), which may be due to similar reasons discussed previously.

S3 Table. Performance of TranSynergy integrated with Graphical Neural Fingerprint (TranSynergyGNF). Extra drug features, graphical neural fingerprint for drug A and drug B extracted with a GCN layer, were concatenated with the output of the Transformer and input into the last fully connected component. The architecture is shown in S2 Fig.

Model	Drug feature	Cell line feature	Pearson	Spearman
TranSynergyGNF	Drug target + chemical structure	Gene dependecies	0.739±0.013	0.722±0.011
		Gene expression	0.730±0.015	0.716±0.022

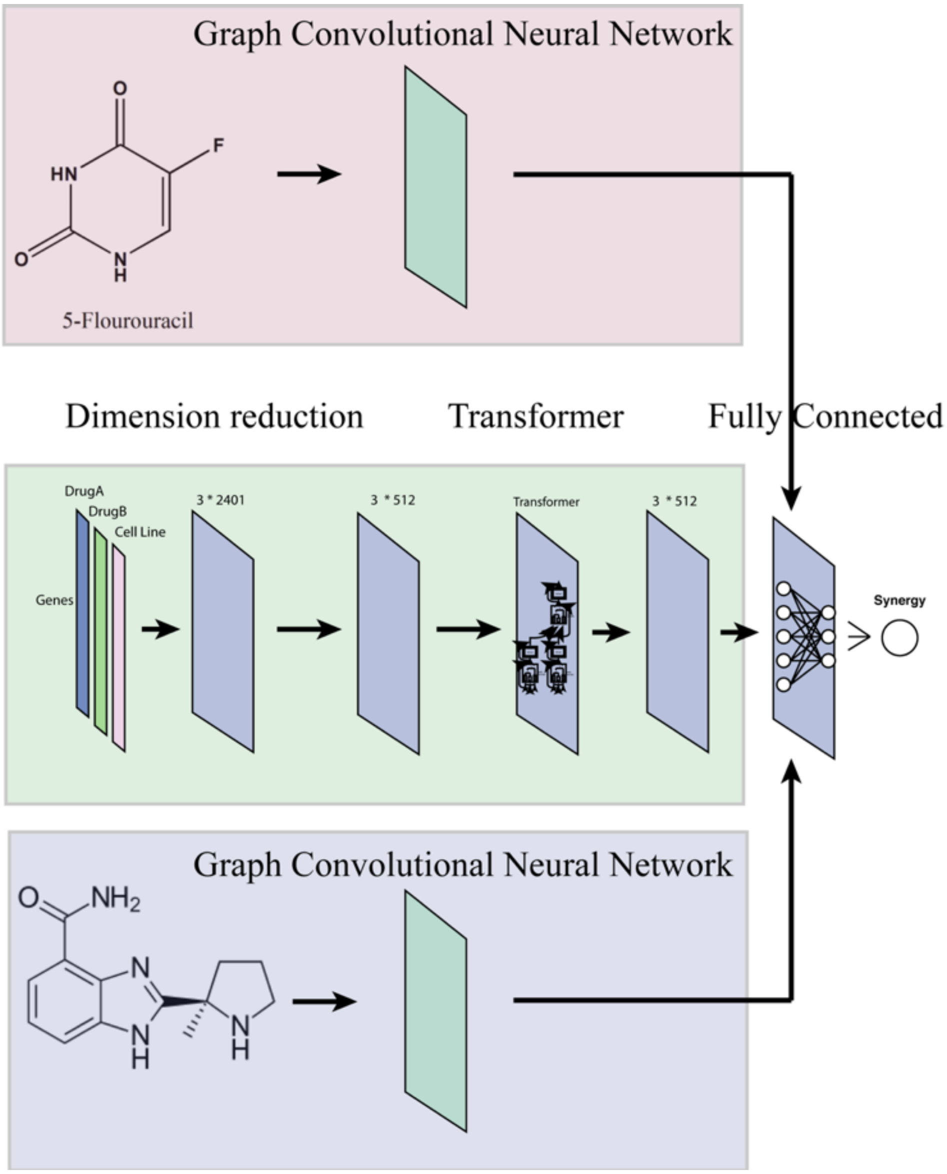

S2 Fig. The architecture of TranSynergy integrated with Graphical Neural Fingerprint (TranSynergyGNF). Extra drug features, graphical neural fingerprint for drug A and drug B extracted with a GCN layer, were concatenated with the output of the Transformer and input into the last fully connected component.

Both new methods failed to boost model performance by integrating chemical information. This might indicate that adding extra chemical information didn't necessarily increase the prediction accuracy in this experimental setup. All the aforementioned results were collected in leave drug combination out scenario (Fig 3A).

The deepsynergy paper uses more robust cross validation strategies than presented in this paper. Why wasn't those strategies used in this work?

Thanks for the comments. To address this comment, we applied nested stratified cross validation method to test the model performance. We added following procedures to the manuscript to clarify this point. .

“Model evaluations were performed in three scenarios. a) In the leave drug combination out scenario, we applied the nested cross-validation method. The data was split into five folds such that the drug pair in one fold does not overlap with the drug pairs in other folds (Fig 3A). Because the drug combination of drug A with drug B and drug B with drug A should have the same drug combination synergy score, the size of training data was doubled by swapping the drug A and drug B. The model was iteratively trained/validated in four folds and then tested in the remaining one fold. b) We further tested our models in the most challenging leave cell out scenario. We used affinity propagation methods to group the cell lines into 4 clusters based on cell line features similarity (Fig 3B). Since 28 cell lines are in the biggest cluster, we used them as the training/validation dataset and held out the remaining cell lines in other clusters as the test dataset (Fig 5). In this way, the cell lines in the testing set are significantly different from those in the training/validation set. c) In the leave drug out scenario, we grouped the drugs into 5 clusters with the affinity propagation method based on their structural similarities (Fig 3C and 6). We chose drugs in one cluster and held out all drug pairs including these three drugs as the test set. The remaining drug pairs are the training/validation set. We performed three tests with keeping out the drugs in each of the three smallest clusters. In each test, the ratio of samples in the training and the testing data is around 4:1.”

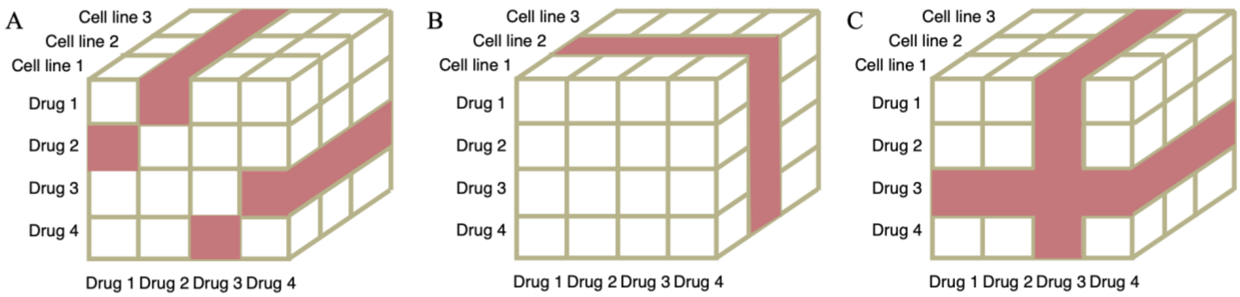

Fig 3. Illustration of three training/test dataset splitting scenarios. Each data point is composed of two drugs and a cell line. The data samples in white and red are the training/validation dataset and test dataset, respectively. A) Leave drug combination out scenario. In this example, drug pairs, including drug1+drug2 and drug_3+drug4, are colored red and held out as test dataset. B) Leave cell line out scenario. In this example, the cell line 2 is colored red and held out as test dataset. C) Leave drug out scenario. In this example, the drug 3 and all drug pairs including the drug 3 are colored red and held out as test dataset.

There are numerous grammatical errors present throughout the paper.

Thanks for the suggestions. We have reviewed our manuscript and corrected many grammar errors.

Reviewer 3:

1> The use of targets to featurize drugs, although helping model interpretability, limits the applicability of the model. Unlike DeepSynergy that only assumes chemical information of the drugs themselves, TranSynergy demands the drug-target association whereas such data is not always available to drugs or compounds. Meanwhile, just using the new compound features with neither new cell line features nor self attention (Ablation Study) led to similar performance as DeepSynergy.

We completely agree with the reviewer that the use of drug target information has limitations for novel drugs whose molecular targets are unknown. This limitation can be partially overcome by using recently developed methods for the accurate prediction of drug targets, e.g. [1]. In addition, drugs used in the drug combination are mainly existing drugs whose targets are largely known, at least, it is true in our benchmark. Because the drug target provides information on the mechanism of drug action, it not only improves the accuracy of the machine learning model for the prediction of synergy but also facilitates the model interpretation. We believe it is beneficial to use drug target features. We have added this discussion in the revision. In addition, we performed additional experiments to use chemical information directly as features. As shown in S1 and S2 Fig., and S2 and S3 Table, the direct use of either chemical descriptors that are the same as DeepSynergy or neuro-fingerprint does not improve the model performance.

2> Please include more details about what DeepSynergy model was used and how it was derived. For fair comparison, DeepSynergy is supposed to be re-trained using the same training set (the original study used cross validation instead) and the training procedures ought to be provided as well.

Sorry that we did not make it clear in the original version. We applied the nested cross-validation method to test the model performance. The same training set and testing set was used and retrained with the code provided in *https://github.com/KristinaPreuer/DeepSynergy*. The details about the procedures are as follows and modified in the manuscript.

“Model evaluations were performed in three scenarios. a) In the leave drug combination out scenario, we applied the nested cross-validation method. The data was split into five folds such that the drug pair in one fold does not overlap with the drug pairs in other folds (Fig 3A). Because the drug combination of drug A with drug B and drug B with drug A should have the same drug combination synergy score, the size of training data was doubled by swapping the drug A and drug B. The model was iteratively trained/validated in four folds and then tested in the remaining one fold. b) We further tested our models in the most challenging leave cell out scenario. We used affinity propagation methods to group the cell lines into 4 clusters based on cell line features similarity (Fig 3B). Since 28 cell lines are in the biggest cluster, we used them as the training/validation dataset and held out the remaining cell lines in other clusters as the test dataset (Fig 5). In this way, the cell lines in the testing set are significantly different from those in the training/validation set. c) In the leave drug out scenario, we grouped the drugs into 5 clusters with the affinity propagation method based on their structural similarities (Fig 3C and 6). We chose drugs in one cluster and held out all drug pairs including these three drugs as the test set. The remaining drug pairs are the training/validation set. We performed three tests with keeping out the drugs in each of the three smallest clusters. In each test, the ratio of samples in the training and the testing data is around 4:1.

In all the above scenarios, the MSE was used as the training loss. We then investigated the Spearman correlation and Pearson correlation between predicted data and ground-truths. ROC-AUC and PR-

AUC were also used to evaluate the model performance for the classification task. Drug pairs with synergy scores larger than 30 were classified as positive pairs and those with lower synergy scores were classified as negative pairs."

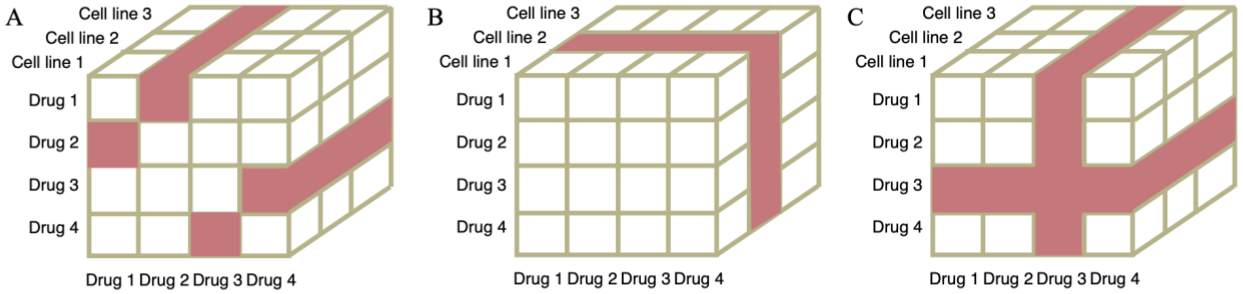

Fig 3. Illustration of three training/test dataset splitting scenarios. Each data point is composed of two drugs and a cell line. The data samples in white and red are the training/validation dataset and test dataset, respectively. A) Leave drug combination out scenario. In this example, drug pairs, including drug1+drug2 and drug_3+drug4, are colored red and held out as test dataset. B) Leave cell lines out scenario. In this example, the cell line 2 is colored red and held out as test dataset. C) Leave drugs out scenario. In this example, the drug 3 is colored red and held out as test dataset.

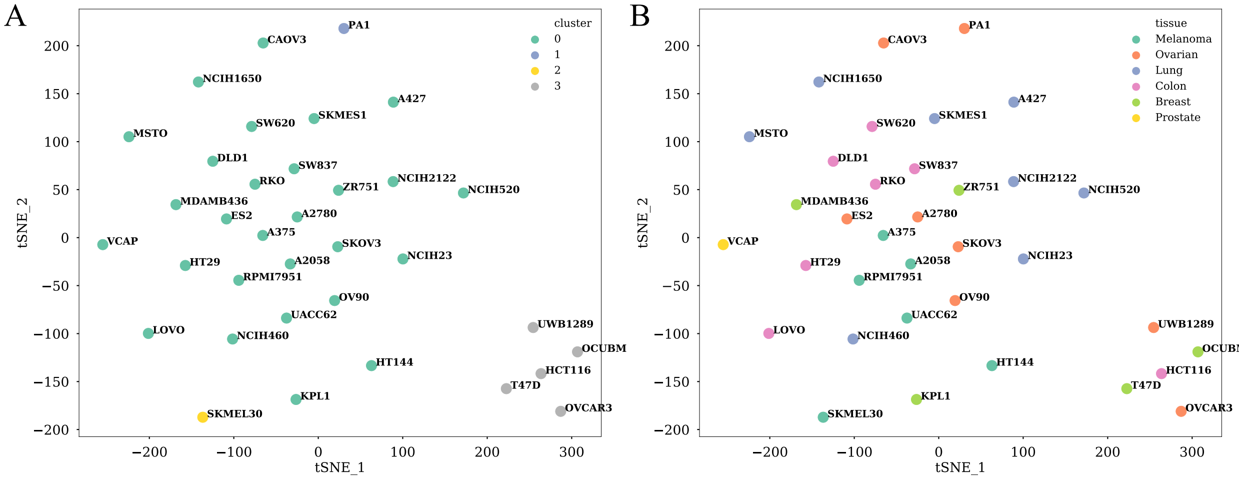

Fig 5. Visualization of different cell lines with t-SNE analysis. High dimensional cell lines vector representations are projected into 2d space with the first two t-SNE components. A) Different colors indicate assigned clusters of each cell line by the affinity propagation method. B) Different colors indicate different tissues of each cell line.

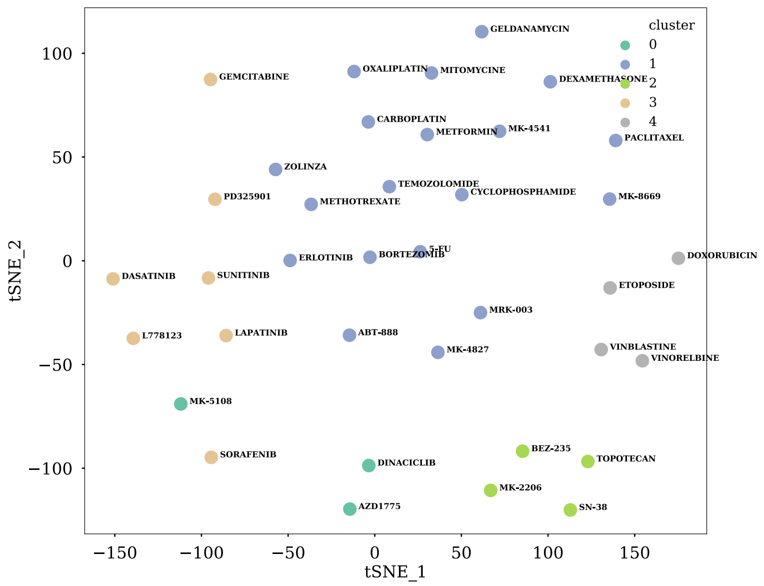

Fig 6. Visualization of drug features with t-SNE analysis. High dimensional cell lines vector representations are projected into 2d space with the first two t-SNE components. Different colors indicate assigned clusters of each drug by affinity propagation method.

3> Is it possible to examine the generalizability of the model under various scenarios of the test set, including new drugs (one or both in the pair), new targets (one or both in the pair), and new cell lines not seen in the training set. Performances on a new dataset (for instance, part of DrugComb) with such scenario splits would be useful. If not, performances on the existing test set with such split could help as well.

Thanks for the suggestions. We agree with the reviewer that the generalizability of the model is an important property to explore for our model. As the reviewer suggested, we checked the model generalizability in the most challenging scenarios, including leave drug out and leave cell line out. We added the detailed procedure into our manuscript, “1) We additionally tested our models in the most challenging leave cells out scenario (Fig 3B). We used affinity propagation methods to divide the cell lines into 4 clusters based on cell line features. Since 28 cell lines are in the biggest cluster, we used them as the training dataset and held out the remaining cell lines as the test dataset (Fig 5). In this way, the cell lines in the test dataset are the most different from those in the training dataset. All the cell lines have a similar amount of data (S4 Table). This is a robust method to test the generalization power of the models. As shown in Table 3 and 4, the performance of the TranSynergy is slightly better than DeepSynergy but both are lower than the first scenario. 2) Similar to the leave cell out scenario, we split the drugs into 5 clusters with the affinity propagation method in leave drug out scenario. We chose drugs in one cluster and held out all drug pairs including those selected drugs as the test set (Fig 3C). The remaining drug pairs are the training/validation set. In this scenario, TranSynergy significantly outperformed DeepSynergy but both were lower than the first two scenarios (Table 3 and 4). ” This is a common out-of-domain problem in machine learning, which could be improved by applying transfer learning strategies. This will be our future direction.

Table 3. Performance of TranSynergy and DeepSyenrgy on leave cells out scenario and leave drugs out scenario.

Model	Leave cells out		Leave drugs out	
	Spearman Correlation	Pearson Correlation	Spearman Correlation	Pearson Correlation
TranSynergy	**0.552 ± 0.013**	**0.513 ± 0.019**	**0.477 ± 0.040**	**0.457 ± 0.031**
DeepSynergy	0.547 ± 0.010	0.501 ± 0.010	0.453 ± 0.025	0.437 ± 0.020
Statistical significance (p-value)	0.0716	0.0013	0.0032	0.0001

4> The performance analyses on different tissues are very interesting and worthwhile further interpretation. To help understand model behaviour, what could have made predictions be high mean and high variance for ovarian cell lines whereas low(er) mean and low variance for prostate cell lines?

Thanks for the comments. The means and variances of tissue specific performance are calculated from the performance of cell lines belonging to each tissue. We suggested that cell line feature differences within each tissue might affect the variances. 8 cell lines belonging to ovarian tissues and only 1 cell line, VCAP, belonging to the prostate cell line. To reflect relationships between cell lines, we performed the t-SNE analysis to visualize the high dimensional vector representation of cell lines mapped to a 2D space with the first two t-SNE components (Fig 5). It showed that the VCAP cell line is located on the edge of one cluster, which indicates the VCAP cell line might have distinctive features. We also noticed that five cell lines were isolated from the remaining cell lines in the t-SNE plot. They included T47D and OCUBM from the breast tissue, HCT116 from the colon tissue, as well as UWB1289 and OVCAR3 from ovarian tissue. The variances of these three tissues were higher than those of the other three tissues. These larger variances among cell line features within these three tissues might contribute to the observed larger performance variances.

5> Interpretation of the model predictions.

a. Self attention was mentioned for encoding drug-target interactions on Page 9 but for representing gene-gene interactions on Page 11. Is there a conflict or the two statements are equal because drugs are represented as target genes (and others in biological networks)?

Thanks for the comments. The one on page 9 should be an error. The self-attention layer intuitively represents gene-gene interactions. We have had that corrected.

b. Moreover, why not use learned self-attentions to interpret the model behavior but use the post-hoc Shapley Additive Gene Set Enrichment Analysis? Or maybe self-attentions were indeed used (although not documented in the enrichment analysis part of the methods)?

The self-attention layer is used to intuitively represent gene-gene interactions and is not used for gene set enrichment analysis. Another reason that we didn't use self-attentions for the interpretation is that previous work has shown that the self-attention learned attention weights may not provide a meaningful explanation for the final prediction [2].

c. The interpretability results emphasize "pathways" associated with the synergy. But the results seem more about genes. It is not clear that pathway information was used for associated pathways, after finding those genes. Also, conceptually, could any explanation be introduced about what pathways are associated with synergy? The same pathway for both drugs? Two overlapping or cross-talking or nonoverlapping pathways covering the same disease module (consistent with the cell line)? Or something else?

Thanks for the comments. The pathways used in GSEA analysis are gene sets that represent oncogenic signatures of cellular pathways, which are often dysregulated in cancer. The GSEA analysis was performed against 189 oncogenic signature gene sets. Those gene sets are named after driver genes, either the key genes or the disrupted known cancer genes for the pathway [3]. For example, "oncogenic signature for RAF overexpressed cells" is the genes dysregulated in cells that stably over-expressing constitutively active RAF1 gene. To clarify this issue, we also changed “pathways” to “oncogenic signature gene sets” in the manuscript.

Regarding how pathways cross-talk interactions contribute to synergy, computational predictions from this work alone cannot provide solid explanations. There is evidence showing that synergy could be explained by any one of the possible scenarios mentioned by the reviewer, pathway cross-talking [4], nonoverlapping pathways [5], or same pathways. A more conclusive statement might require additional experimental evidence, which is out of our work’s scope. We have added this discussion in the revision.

Minor comments:

6> Please include more details about the 3650 novel samples in the section of methods.

Thanks for the suggestions. As suggested by reviewer, we added the following descriptions to the manuscript. “Even though the majority of drug combinations composed of the drugs available in this study are explored experimentally [6], here are still 3650 novel drug pairs that haven’t been tested.”

7> With the current readme of the github repo, it is hard to figure out the structure of many source code files laid at the same top level. In addition, to help achieve more impact of the work, please consider including in readme how to train the model with the codes and which data files are what.

Thanks for the suggestions. We have reorganized GitHub layout and added more descriptions to the out GitHub about the data files.

8> There were light grammar issues.

Thanks for the suggestions. We have reviewed our manuscript and corrected many grammar errors.

1. Cai T, Lim H, Abbu KA, Qiu Y, Nussinov R, Xie L. A deep learning framework for elucidating whole-genome chemical interaction space. bioRxiv. 2020:2020.10.08.332346. doi: 10.1101/2020.10.08.332346.
2. Jain S, Wallace BC. Attention is not Explanation. 2019.

3. Subramanian A, Tamayo P, Mootha VK, Mukherjee S, Ebert BL, Gillette MA, et al. Gene set enrichment analysis: a knowledge-based approach for interpreting genome-wide expression profiles. Proc Natl Acad Sci U S A. 2005;102(43):15545-50. Epub 2005/10/04. doi: 10.1073/pnas.0506580102. PubMed PMID: 16199517; PubMed Central PMCID: PMCPMC1239896.
4. Jaeger S, Igea A, Arroyo R, Alcalde V, Canovas B, Orozco M, et al. Quantification of Pathway Cross-talk Reveals Novel Synergistic Drug Combinations for Breast Cancer. Cancer Res. 2017;77(2):459-69. Epub 2016/11/24. doi: 10.1158/0008-5472.CAN-16-0097. PubMed PMID: 27879272.
5. Cheng F, Kovacs IA, Barabasi AL. Network-based prediction of drug combinations. Nat Commun. 2019;10(1):1197. Epub 2019/03/15. doi: 10.1038/s41467-019-09186-x. PubMed PMID: 30867426; PubMed Central PMCID: PMCPMC6416394.
6. O'Neil J, Benita Y, Feldman I, Chenard M, Roberts B, Liu Y, et al. An Unbiased Oncology Compound Screen to Identify Novel Combination Strategies. Mol Cancer Ther. 2016;15(6):1155-62. Epub 2016/03/18. doi: 10.1158/1535-7163.MCT-15-0843. PubMed PMID: 26983881.
